# Intensive Interventions for Burnout, Stress and Occupational Well-Being: A Critical Review and a Delivery-Format Framework

**DOI:** 10.3390/jcm15207887

**Published:** 2026-10-13

**Authors:** Anne Marie Novak, Shahar Lev-Ari

**Affiliations:** 1 School of Public Health, Gray Faculty of Medical and Health Sciences, Tel Aviv University, Tel Aviv 69978, Israel; annemarie@tauex.tau.ac.il; 2 Department of Genetics & Center on Stress and Health, School of Medicine, Stanford University, Stanford, CA 94305, USA

**Keywords:** burnout, occupational well-being, immersive interventions, retreats, intensive, workshop, bootcamp, virtual program, review

## Abstract

Intensive programs such as retreats and workshops are widely promoted for occupational burnout, but their evidence base is limited and fragmented. This critical narrative review searched PubMed, Google Scholar, and APA PsycInfo for peer-reviewed studies published from January 2000 to March 2026. Eligible studies evaluated structured, high-dose interventions delivered in a condensed format, reported burnout or closely related outcomes, and involved adult working or occupationally relevant training populations. Evidence was organized by four immersion dimensions: contextual containment, dose density, experiential multimodality, and social density. Forty studies met the criteria: 22 residential retreats and 18 non-residential intensives. No virtual synchronous program qualified, so adjacent virtual evidence was appraised narratively. Studies commonly reported short-term gains in stress and well-being and high satisfaction, but effects on validated burnout measures were less consistent. Most studies were small, uncontrolled, and drawn from healthcare and student samples, with little long-term follow-up; controlled trials yielded more conservative results. No study compared the same curriculum across formats. Delivery format may itself be an active ingredient, with facilitated social connection the best-supported candidate. Three testable pathways are proposed: contextual containment as a reset for exhaustion, shared group experience and communitas for disengagement and cynicism, and dose density as a catalyst for professional fulfillment and efficacy. Head-to-head format comparisons, standardized burnout measures, longer follow-up, and economic evaluation are needed.

## 1. Introduction

Burnout is a work-related syndrome marked by debilitating exhaustion, distancing from work, and often reduced professional efficacy and fulfillment. The World Health Organization classifies burnout as an occupational phenomenon caused by chronic workplace stress that has not been successfully managed. It defines three dimensions: energy depletion or exhaustion; increased mental distance, disengagement, negativism, or cynicism toward one’s job; and reduced professional efficacy [1]. Occupational well-being theories locate burnout in prolonged imbalance: high job demands persist while resources and recovery remain insufficient, allowing strain to accumulate [2,3,4]. The consequences extend beyond reduced satisfaction and performance. Prospective evidence links burnout to adverse physical and mental health outcomes, sickness absence, and work disability [5], and identifies it as an independent risk factor for coronary heart disease [6], type 2 diabetes [7], and musculoskeletal pain [8]. The economic burden is substantial, with burnout-related turnover and reduced work hours estimated to cost employers billions of dollars each year [9].

A large evidence base has examined burnout interventions, especially among healthcare workers [10,11,12]. Meta-analyses suggest that individual- and organization-directed programs can help, but their effects are usually small to modest and hard to sustain. Organization-directed approaches may be more durable [13,14], supporting the view that burnout arises from working conditions rather than individual weakness. Yet workplaces often struggle to implement structural change [15]. At the same time, many workers seek self-help [16] but lack the capacity for multi-week courses or gradual programs that require sustained attendance. This implementation gap, together with popular interest in self-optimization and mental health [17], has helped fuel the growth of short, high engagement, “immersive” formats: residential retreats, weekend intensives, seminars, workshops, and bootcamps delivered in person or through synchronous online platforms. Their appeal follows from current burnout models: if burnout reflects prolonged exposure, depleted resources, and inadequate recovery, a protected “reset” may restore resources and redirect behavior [2,18,19]. Whether these interventions reliably improve burnout, and under what conditions, remains underexplored.

Research to date has centered mainly on residential retreats, often in wellness tourism, lifestyle modification, and spiritual or condition-specific programs. Retreats are structured, live-in programs in which participants leave daily routines, stay together at a facility such as a hotel or wellness center, and participate in classes, workshops, and group sessions while sharing meals and communal space. They may last from a few days to several weeks or months. Depending on the program, they aim to provide rest, focused development, or intensive healing in a low-distraction environment. A systematic review of residential retreats reported improvement in at least one outcome in every included study, spanning stress, anxiety, depression, quality of life, and physiological markers; some effects persisted for months to years [20]. The review also exposed major limits: heterogeneous interventions, weak methods, rare adverse-event reporting, and almost no economic evaluations [20]. A key interpretive problem is that contextual removal and recovery may themselves drive benefit. Vacation research shows small-to-moderate gains during time away, often followed by rapid fading after a return to routine, making it hard to separate “content” from “context” [21]. Vacations that include meditation can yield greater medium-term gains than ordinary vacations, although intensive retreat meditation may differ little from lower-dose vacation meditation. This reinforces the need to specify active ingredients beyond “time away” [22]. Non-residential single-day or multi-day intensives have also emerged [10,11,12], offering similar curricula without shared living.

The delivery format itself has begun to attract attention as a possible active ingredient. In one comparison of an immersive seminar or workshop with a traditional lecture, immersive participation produced greater physiological arousal, higher self-reported engagement, and greater perceived impact [23]. Immersion may therefore shape engagement and learning, which is highly relevant for depleted, disengaged participants. Immersive formats may work through mechanisms that low-dose weekly programs or online modules struggle to activate: attentional capture and engagement [23], contextual shift and recovery opportunity [20,21], and social processes that accelerate motivation and support [24]. Durability remains the central problem. Behavior-change science distinguishes initiation from maintenance and emphasizes that lasting change depends on self-regulation and environmental supports [25]. Because habits typically form over weeks to months, brief intensives may need post-program scaffolding to convert initial gains into durable patterns [26].

These observations call for a synthesis that treats delivery format as part of the intervention. Immersive interventions are increasingly common and commercially prominent [17], yet their evidence remains fragmented across disciplines. This review clarifies definitions, distinguishes delivery formats, summarizes evidence for burnout and related constructs, and identifies key knowledge gaps.

## 2. Materials and Methods

We conducted a critical narrative review to map and appraise evidence on intensive interventions for burnout. We did not pool effects quantitatively. We searched PubMed, Google Scholar, and APA PsycInfo for peer-reviewed articles published from January 2000 through March 2026, with supplementary web sources cited for commercial market context in the Discussion section accessed during manuscript preparation in April 2026. A complete description of the literature-search and study-selection procedures, including reproducible database-specific search strategies and the process for handling reviewer disagreements, is available in the Supplementary Materials. The search combined population and outcome terms (burnout, stress, well-being, exhaustion, cynicism, disengagement, professional, occupational, job-related, work, fulfillment, resilience) with format terms (immersive, intensive, retreat, workshop, course, bootcamp, seminar, group, virtual, synchronous, hybrid, online). We included studies that evaluated structured, high-dose interventions (at minimum a single half-day block, or consecutive days) delivered in a condensed timeframe, reported burnout or closely related outcomes, and involved adult working populations or occupationally relevant training populations. We excluded low-dose longitudinal programs (for example, standard 8-week MBSR), interventions for specific clinical conditions (such as diabetes camps), interventions using medications, herbs, or other substances, and recreational tourism without a structured curriculum. Multi-arm studies were eligible when at least one arm met the temporal-compression criterion. Primary studies extracted for analysis in this review were cross-checked against the underlying studies included in cited systematic reviews and meta-analyses. Systematic reviews are cited strictly to provide broad context, while primary studies are evaluated individually for their specific occupational burnout and stress metrics.

Because this manuscript was conducted as a critical narrative review rather than a quantitative systematic review, a formal risk of bias assessment was not performed. Instead, methodological limitations of the included studies, such as uncontrolled designs and small sample sizes, are narratively appraised throughout the review.

Screening yielded 40 studies: 22 residential retreat studies (Table 1) and 18 non-residential in-person intensives (Table 2). No identified virtual study met the inclusion criteria, and virtual synchronous programs are strictly classified as adjacent conceptual evidence rather than qualifying studies. These are discussed narratively to approximate dismantling evidence. To map the complete delivery format framework, we extended our search to ClinicalTrials.gov to identify registered, ongoing or unpublished trials of synchronous virtual intensives. Section 6 provides a formal appraisal of this empirical gap in the published literature and evaluates these pipeline virtual studies. We include this adjacent literature because it helps isolate which immersion dimensions survive when physical containment is removed and because it contains some of the most rigorously controlled trials in the field. Virtual studies approximate dismantling evidence by removing one or more immersion dimensions while holding curriculum broadly constant. Section 6 therefore uses virtual evidence to motivate the proposed framework; it does not treat virtual programs as qualifying studies, and no evidence table is provided for them.

The authors designed the three conceptual figures by specifying their content, structure, and layout. Generative AI tools (Canva AI and Napkin AI) rendered these author-specified designs into finished graphics. The authors reviewed and verified all figures and take full responsibility for their content. Grammarly, including its generative rewriting features, was used to edit and rephrase author-written text for clarity and language. The authors developed all content, arguments, and interpretations.

## 3. Defining Intensive Interventions: A Working Framework

Because academic and commercial sources use “immersive”, “intensive”, “bootcamp”, “workshop”, and “retreat” inconsistently, this review adopts a pragmatic, delivery-based definition: intensive interventions are structured, group-based programs that deliver a high dose of experiential content within a condensed time window through sustained synchronous engagement. To qualify as an intensive intervention for this review, a program must meet the following temporal parameters: (1) Continuous Temporal Compression: The primary intervention curriculum must be delivered as a single continuous block (minimum half a day, or 4 h) or across consecutive days without multi-day gaps between core sessions. (2) Non-Distributed Delivery: The foundational curriculum cannot be distributed incrementally over weeks or months (e.g., standard 8-week MBSR). Programs incorporating post-intervention booster sessions or follow-up digital nudges remain eligible, provided the primary educational or experiential dose is delivered in the initial compressed block.

Immersion has four interrelated dimensions: (1) dose density, or temporal compression into a single day or multiple consecutive days; (2) contextual containment, or removal from routine demands and everyday environments; (3) experiential multimodality, or practice-based use of multiple modalities rather than didactic content alone; and (4) social density, or guided group process. These dimensions create a spectrum. Residential retreats sit high on all four: they deliver a multi-day block in a setting removed from routine, often with full-body or nature-based practice and shared living. Non-residential intensives maintain dose and social density but only partly separate participants from daily life because participants do not share lodging. Virtual synchronous programs preserve live group contact but provide the weakest containment and the most screen-mediated, variably dosed experience. Accessibility moves in the opposite direction: lowest for residential retreats and highest for virtual delivery. The three formats therefore differ in mechanisms, equity, scalability, and risk.

## 4. Residential Retreats

Residential retreats occupy the highest-containment end of the immersive spectrum. They combine removal from routine demands, environmental change, nature exposure, digital restriction, and simplified schedules. They also create unusually high social density: participants share lodging, meals, and facilitated group work over several days or weeks. Within the Job Demands–Resources framework [2], this format may target burnout through two routes: restoration of depleted recovery resources through protected time away, and accelerated acquisition of psychological and behavioral skills (such as mindfulness, emotion regulation, self-compassion, and interpersonal skills) that may support re-engagement with work after return. Retreats also create a liminal context that suspends role expectations and habitual cues, enabling reflective reappraisal, identity-level shifts, and intensified social learning. The same containment, however, creates the central interpretive problem of the retreat literature. Improvements may reflect the “vacation effect” (contextual removal and lifestyle simplification), as much as curriculum. Durability depends on whether a brief, protected reset survives the return home.

Table 1 summarizes the 22 residential retreats that met the inclusion criteria and were most relevant to burnout and stress in adult working populations. Overall, retreat studies vary widely in population, program type, and outcome, but their findings point in a broadly consistent direction. Naidoo and colleagues synthesized 23 residential retreat studies (1995–2017) across diverse modalities and found that every study improved at least one outcome, from psychological symptoms and quality of life to physiological markers [20]. Still, the evidence was methodologically mixed, adverse events were rarely reported, and economic evaluations were largely absent [20]. A focused meta-analysis of mindfulness meditation retreats in non-clinical adults reached a similar conclusion: medium-to-large pre-post improvements in stress, anxiety, depression, and mindfulness, alongside substantial heterogeneity and generally low study quality [48]. Design limitations and inconsistent follow-up continue to constrain causal attribution and mechanism identification.

Several healthy-adult studies show that short residential retreats can shift stress markers and outcomes indirectly related to burnout while illustrating the content-versus-context problem. Vacation-at-the-same-venue controls offer one strategy for separating retreat content from contextual recovery. Epel and colleagues randomized healthy women to a 6-day meditation and yoga retreat or an on-site vacation control, with an additional non-randomized group of experienced meditators [39]. Large transcriptome-wide changes appeared across all groups, suggesting that contextual removal, rest, and resort-based lifestyle change can drive robust short-term molecular signatures. The physical setting itself may contribute to this downshifting: immersive nature experiences such as forest bathing and wilderness therapy have been linked to changes in cortisol and heart-rate variability [69]. Psychological well-being also improved across groups. Retreat participants showed more durable gains in depressive symptoms and stress at 10 months than the vacation group, and experienced meditators showed distinct cellular-aging signatures, including increased telomerase activity [39]. Gardi and colleagues randomized healthy adults to a 3-day residential mindfulness retreat or an active residential vacation control [40]. Both groups reduced perceived stress, consistent with a general recovery effect, but the retreat produced greater reductions in anxiety and stress-related biomarkers (cortisol, IL-6, IL-8) and greater increases in mindfulness and anti-inflammatory IL-10. Attention restoration theory offers one explanation for the nature-retreat arm, proposing that natural environments support effortless attentional recovery and reduce cognitive fatigue [38].

Sequential crossover designs can help determine whether different contemplative curricula add distinct benefits within the same immersive container. Zangrì and colleagues delivered intensive Mindfulness-Based Stress Reduction and Compassion Cultivation Training in randomized order within a 7-day retreat, with assessments during the retreat and at 3 months [47]. Participants improved in stress, emotion regulation, self-compassion, mindfulness, and affect, with some order effects favoring mindfulness-first sequencing for maintenance. The study also monitored adverse effects, an important but rare practice given the risks of sleep disruption and emotional sensitivity in intensive contemplative contexts. Emerging protocols that integrate multi-level endpoints (EEG, epigenetics, cortisol) reflect a “retreat-as-laboratory” paradigm, but they have not yet contributed outcome evidence [70].

Direct evidence linking residential retreats to burnout in working adults is thinner than the broader stress and well-being literature. Several occupationally relevant studies suggest benefits on proximal burnout pathways such as stress, sleep, resilience, self-compassion, and work engagement, and in some contexts, on burnout dimensions or professional fulfillment. Bailey and colleagues evaluated a two-day residential “Intentional Practitioner Retreat” for physicians and advanced practice providers using a pre–post design [27]. Perceived stress fell and professional fulfillment improved immediately after the retreat, while other outcomes changed little and follow-up data were sparse. Stronger follow-up is needed to evaluate durability. The largest cluster of occupational retreat evidence comes from the RISE program (Resilience, Integration, Self-awareness, Engagement), a yoga- and mindfulness-based residential retreat delivered in 3- or 5-day formats across professional groups [29,30,35,36,45]. This program has been evaluated across distinct, non-overlapping participant cohorts rather than through repeated analyses of a single population. These independent evaluations span an initial pilot with general frontline professionals [45], a pragmatic controlled trial of frontline workers [36], and sequential cohorts of rural educators [29,35] and urban education administrators [30]. Across these distinct cohorts, RISE participation has been consistently associated with improvements in stress, mindfulness, self-compassion, empowerment, affect, sleep quality, and work engagement, some maintained at two months [29,30,35,36,45].

Burnout-specific outcomes are less consistent. Dyer and colleagues found no significant between-group changes in MBI-GS dimensions in a small pragmatic controlled design, and Trent and colleagues reported marginal improvements in exhaustion, with no clear change in cynicism or professional efficacy, in a non-randomized controlled trial [29,36]. In contrast, a single-arm study in education professionals reported follow-up improvements in professional efficacy, and in some analyses, exhaustion [35]. These differences may reflect baseline burnout severity, occupational context, and the possibility that retreats move proximal determinants like sleep, emotion regulation or self-compassion more readily than work-embedded dimensions such as cynicism and disengagement, which may require organizational change or sustained post-retreat support. Reeves and colleagues interviewed RISE participants three months later and found continued skill use, greater “permission” for self-care, and workplace spillover [71]. This pattern fits behavior-change models: retreats may catalyze motivation and self-efficacy, but durable occupational change depends on habit formation, social reinforcement, and environmental support.

Two further studies extend the occupational picture. Gold and colleagues evaluated a two-day residential couples workshop for physicians and their partners using a delayed-intervention comparator. At six months, participants showed medium-to-large improvements in relationship strain and burnout-related measures, including work exhaustion and interpersonal disengagement [31]. This study treats burnout as relational and systemic spillover, suggesting that retreats may work partly by repairing social resources that buffer occupational strain. O’Shea and colleagues observed short-term reductions in emotional exhaustion and perceived stress after a department-funded resident retreat with mindfulness training [32], and Schroeder and colleagues found large reductions in emotional exhaustion and depersonalization three months after a brief weekend mindfulness curriculum for physicians [34]. Qualitative and descriptive studies [72,73,74] reinforce a recurring point: participants often report high benefit, but measurement is often too weak to show whether retreats reduce burnout rather than provide valued respite.

**Figure 1:**
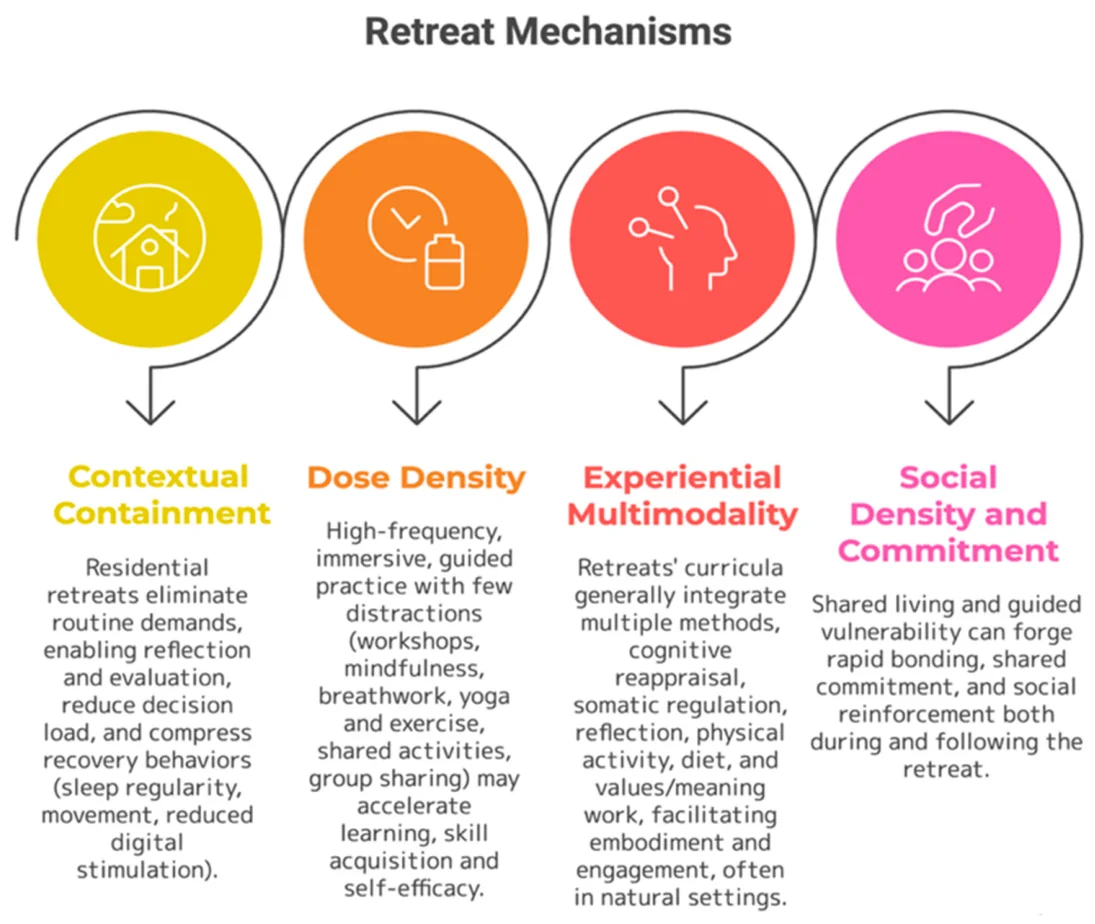
Four candidate mechanisms of residential retreats, mapped to the immersion framework: contextual containment, dose density, experiential multimodality, and social density. Each panel summarizes the immersion feature and the burnout-relevant process it is hypothesized to engage. These four dimensions are consolidated into three change pathways in the Discussion section (Figure 3).

Across modalities, four candidate mechanisms recur and map onto the immersion framework (Figure 1):

a. Contextual containment and recovery opportunity. Retreats remove routine demands, reduce decision load, and compress recovery behaviors such as regular sleep, movement, and reduced digital stimulation. Vacation-control designs suggest that containment produces large short-term psychological and biological shifts. For burnout, this pathway may transiently reduce exhaustion and improve mood; without changes to work conditions, however, it may not address cynicism or professional efficacy.
b. Dose density and skill acquisition. High-frequency guided practice with few distractions, including modalities like mindfulness, breathwork, yoga, and compassionate attention, may accelerate learning and self-efficacy. Sustained increases in mindfulness and self-compassion at follow-up are consistent with retreats initiating skills that consolidate over weeks when practice continues.
c. Experiential multimodality and embodied regulation. Retreats integrate cognitive reappraisal, somatic regulation, interpersonal reflection, physical activity, and values or meaning work. Qualitative accounts emphasize embodiment and affect, supporting the view that burnout recovery may require not only cognitive insight, but also physiological downshifting and renewed pleasure, connection, and agency.
d. Social density and facilitated group process. Shared living and guided vulnerability can create rapid bonding, shared commitment, and social reinforcement. Social-resource restoration may be especially relevant to disengagement, cynicism, and professional meaning; retreats structured around camaraderie or relational repair suggest that these mechanisms may be central.

Table 1 summarizes the 22 residential retreats that met the inclusion criteria and were most relevant to burnout and stress in adult working populations [22,27,28,29,30,31,32,33,34,35,36,37,38,39,40,41,42,43,44,45,46,47]. The table is subdivided into studies providing direct evidence (evaluating occupational burnout using validated instruments) [27,28,29,30,31,32,33,34,35,36] and those providing indirect evidence (evaluating stress, stress-related biomarkers, and general well-being) [22,37,38,39,40,41,42,43,44,45,46,47].

Among the standardized retreat formats, Vipassana silent meditation retreats offer a well-defined high-containment model. Participants keep silence, surrender digital devices and outside contact, follow simplified schedules, and meditate 8 to 10 h a day, typically for 10 consecutive days. This structure delivers near-total contextual containment and high dose density, although silence suppresses the peer sharing and facilitated discussion that drive many other retreat formats.

Two systematic reviews have synthesized Vipassana-specific evidence. Chiesa (2010) found seven studies, mostly of poor methodological quality, and called for more rigorous designs [75]. Giridharan et al. (2025) reviewed 11 studies (three RCTs, two single-arm trials, and five observational studies; total *N* = 792, median *N* = 40) and reported moderate evidence for reduced stress and anxiety, higher mindfulness and well-being, and physiological change, including improved heart-rate variability [76]. Effects appeared intensity-dependent, although all three RCTs were rated at high risk of bias and none used blinding [76]. In a stronger quasi-experimental study, Szekeres and Wertheim (2015) used motivationally matched early-enroller controls (*N* = 172) and 6-month follow-up [77]. They found medium-to-large post-course gains in stress, well-being, mindfulness, and self-kindness; by follow-up, stress gains had eroded while mindfulness and self-kindness held. This evidence base is almost entirely disconnected from occupational populations: no study measured validated burnout dimensions in working adults.

In sum, residential retreats are a high-containment intervention family with plausible relevance to burnout through recovery restoration, accelerated skill acquisition, embodied regulation, and social-resource rebuilding. By compressing training into a few days, they may also bypass the high dropout rates of standard multi-week interventions [78,79]. The small but growing evidence base supports short-term improvements in stress-related outcomes, and in some contexts, in professional fulfillment and burnout dimensions. Causal attribution remains difficult because contextual effects, small samples, short follow-up, and variation in content, dose, facilitator training, and nature exposure may each drive physiological recovery [69].

Progress requires better reporting of retreat components and fidelity. Retreats can be resource-intensive and may privilege participants with flexible schedules and financial means, yet economic evaluations and equity-focused recruitment and reporting remain rare. Most studies recruit healthy volunteers and report few occupational metrics. Follow-up is also inconsistent and often short. Studies of working adults should include validated burnout dimensions: exhaustion, disengagement or cynicism, and professional efficacy or fulfillment, along with work-functioning outcomes. Multi-wave follow-up paired with mechanistic measures (sleep, affect regulation, practice adherence, social support, lifestyle, and career outcomes) would clarify how initial gains translate into sustained change and help detect “sleeper effects”, in which newly acquired skills take months to produce durable resilience [42].

## 5. Non-Residential Intensives

Non-residential intensive interventions such as workshops, seminars, “bootcamps” or single-day retreats retain dose density, experiential multimodality, and social density but provide weaker contextual containment. They bring participants together to acquire skills and resources quickly, but the participants then return to routine demands the same day. The format preserves many proposed active ingredients while losing the restorative mechanisms tied to environmental separation. However, its practical appeal is substantial: compared with multi-week programs or costly retreats, non-residential intensives require less time away, lower cost, and less coordination. Emerging evidence suggests that the standard multi-week format may not be necessary for meaningful improvement [56], although direct mechanistic evidence from workshop research remains sparse.

The rationale for brief, high-dose delivery rests on mechanisms that may distinguish it from lower-dose gradual formats. First, high-dose workshops often prioritize somatic and physiological regulation: breathwork, mindful movement, progressive muscle relaxation, and guided body awareness [13]. These practices may create a transient physiological “reset”, lower autonomic arousal, and interrupt the chronic activation that fuels emotional exhaustion. Immersive learning research found that immersive seminars produced greater physiological arousal modulation and enhanced mood than traditional lectures, suggesting that format shapes psychophysiological engagement [23]. Whether this mechanism transfers to non-residential burnout workshops remains untested, although several reviewed studies include somatic techniques aimed at this pathway [49,50,58].

Second, the condensed format may act as a motivational catalyst without the need for an overnight stay and multi-day engagement. Burnout-related exhaustion and cynicism can undermine engagement with traditional, gradual interventions such as weekly therapy; participants may struggle to sustain attention amid ongoing load, contributing to dropout. An immersive block demands attention over hours and may break through mental distance, catalyzing motivation and a sense of possibility. This aligns with behavior-change models that distinguish initiation from maintenance [25]. Still, this mechanism remains speculative: no reviewed non-residential study directly measured motivational-stage transitions or compared engagement trajectories across formats.

Third, non-residential intensives can still create high social density in a compressed period. Facilitated group processes including shared vulnerability, role-play, peer reflection, and collective problem-solving can build bonding and psychological safety similarly to residential retreats and more quickly than weekly drop-in formats. Roy and colleagues found that participants consistently identified shared experience and peer-support tools as the most valued elements [66], and group reflection may normalize distress in caregiving roles and reduce guilt about self-compassion [62]. More broadly, successful burnout interventions may work by enhancing psychosocial mediators such as job control, social support, and participation in decision-making. Non-residential intensives can offer employees a designated space to build these resources collectively, even when they cannot restructure the organizational conditions that depleted them [67].

The identified studies mainly recruited healthcare professionals, trainees, and students in health-related fields, including nursing, medicine, mental health, and occupational therapy. Non-healthcare populations were rare. Many studies involved students [52,80,81,82], who were included because training-related burnout mirrors occupational burnout in its dimensions and consequences; however, this choice limits generalizability to established professionals and other sectors. Most designs were uncontrolled pre–post evaluations. Only a few used randomized or equivalent controls [51,57,60,65], and these produced more conservative findings. Furthermore, several studies relied solely on indirect markers of well-being, recovery behaviors, or perceived stress rather than multidimensional burnout instruments.

With these caveats, evidence shows that non-residential intensives can produce short-term improvements in burnout-related outcomes, especially emotional exhaustion and perceived stress, though the effects are inconsistent and often fade. Among the mindfulness-based formats, Morrow and colleagues compared four variants for healthcare workers: standard 8-week MBSR, a 4-week condensed course, and two single-day intensives. All variants reduced burnout and stress, with roughly 75% higher odds of improved burnout scores and no significant difference between brief and traditional formats; however, the study lacked a control group and had substantial attrition [56]. Long Franco and Christie found that a 1-day adaptation of Self-Compassion for Healthcare Communities reduced burnout, anxiety, and stress in pediatric nurses, with effects maintained at 3 months [55]. Sarazine and colleagues reported reduced perceived stress and emotional exhaustion 1 month after a 4-h mindfulness workshop for nurses; exhaustion gains persisted at 6 months while stress gains faded [59]. Martin and colleagues found increased self-compassion and decreased perceived stress at 2 months in an uncontrolled pilot for health professions students [64].

Single-day CBT and psychoeducation interventions also show promise. A 6-h “Self-Care Strategies for Nurses” program [54], grounded in Lazarus and Folkman’s cognitive model of stress and coping and delivered through psychoeducation, art making, and relaxation and guided imagery practice, significantly reduced emotional exhaustion and depersonalization immediately after the course, but the personal accomplishment findings were reported inconsistently, and there was no follow-up [54]. Alenezi and colleagues found improvements across all three MBI subscales at 1 month after a program for mental health nurses, with effects sustained to 6 months [49].

Other formats produced mixed results. Hill and colleagues found increased personal accomplishment after off-site training, but exhaustion and depersonalization did not change [53]. Interventions that combine individual coping with organizational awareness may be more promising: Żołnierczyk-Zreda’s teacher workshop, based on Karasek’s job-stress model, reduced emotional exhaustion while increasing behavioral job control and reducing perceived intellectual demands and workload [60]. Poulsen and colleagues’ recovery workshop for oncology staff increased recovery behaviors and sleep quality, buffering the longitudinal decline seen in the controls [65]. Amirianfar and colleagues found non-significant improvements across burnout subscales after a 1-day wellness day for rehabilitation trainees, most pronounced at 3 months [50].

However, evidence from controlled studies is less promising, suggesting that uncontrolled studies likely overestimate effects through regression to the mean, expectancy, and lack of a counterfactual. In the most conservative randomized controlled trial of a non-residential workshop in this review, Axisa and colleagues evaluated a 4.5-h well-being workshop for Australian physician trainees. They found no significant improvements in depression, anxiety, stress, traumatic stress, burnout, compassion satisfaction, or alcohol use at 1 or 6 months; small reductions emerged at 6 months but did not reach significance [51]. The 6-h BREATHE workshop (Burnout Reduction: Enhanced Awareness, Tools, Handouts, and Education) produced large reductions in emotional exhaustion and moderate reductions in depersonalization at 6 weeks in an initial, uncontrolled pilot cohort of community mental health providers [58]. However, a subsequent, independent multisite randomized controlled trial randomized a new, distinct cohort of behavioral health providers to BREATHE or an active control [57]. This study found no significant between-group differences in burnout, job satisfaction, turnover intentions, absences, or work–life balance [57].

Many studies on non-residential intensives use uncontrolled, quasi-experimental, or retrospective pre–post designs. As Ahola and colleagues note, individually focused interventions often fail to show statistically significant long-term relief under rigorous RCT standards; high risk of bias, absent active controls, and small samples limit generalizability [11]. High satisfaction alongside weak outcome evidence recurs across other non-residential studies [62,66,83]. Fade-out is pronounced. Awa and colleagues found that although 82% of person-directed interventions reduce distress, effects usually last no more than six months [68]. Initial motivation and physiological relief often erode when participants return to unchanged, high-demand environments. Haber and colleagues’ radiology residency wellness day illustrates another limitation: 97% of respondents expected it to improve their residency, but the study used post-only satisfaction ratings without baseline or validated outcomes [61].

Brief single-session workshops lasting under four hours fall below the half-day intensive threshold and were excluded, yet they offer useful boundary evidence on the lower limits of dose density [80,81,82,83,84]. Implemented mainly in university and health-professions training, these ultra-brief sessions can yield immediate gains in perceived preparedness and resiliency concepts (2 h [83]), short-term improvements in academic stress and mental well-being (3 h [82]), or lower student anxiety over a 4-week follow-up (90 min [84]). However, ultra-brief doses rarely withstand ongoing training demands or controlled comparison. After a 2-h clerkship transition workshop, medical students’ gains in coping confidence vanished by 3 months while burnout increased [80], and a 90-min onsite coping seminar showed only small, non-significant between-group differences compared with an online writing exercise and historical controls, despite favorable within-group trends [81].

Bridging initial relief and durable recovery requires addressing several structural gaps. First, brief intensives need ongoing scaffolding. Because gains rarely persist beyond six months without refresher courses [68], studies should test booster sessions, digital nudges, and other check-ins as ways to convert workshop skills into durable workplace habits. Second, person-directed workshops should be paired with organization-directed change. Systematic reviews increasingly find that combined approaches are most durable because they build individual resources while reducing environmental stressors [67]. Workplace intensives should therefore be paired with workflow redesign, managerial-support training, or protected recovery time to prevent “re-entry shock”. Third, heterogeneous outcome measures hinder synthesis [11]. Studies should use validated burnout instruments (MBI, OLBI, PFI, or BAT) across multiple time points and include objective longitudinal markers such as absenteeism, turnover, and return-to-work. Table 2 summarizes findings from 18 non-residential intensive interventions. The evidence is subdivided into studies evaluating direct occupational burnout outcomes [49,50,51,52,53,54,55,56,57,58,59,60] and those evaluating indirect stress, recovery, or well-being markers [61,62,63,64,65,66].

## 6. Virtual Synchronous Programs

Within this framework, synchronous online programs (virtual workshops and seminars, webinars, and online retreats) trade contextual containment for accessibility. Participants remain in everyday environments, sometimes the same settings that generate occupational stress, while participating in screen-mediated group engagement. Historically, researchers have relied on in-person or residential formats to deliver the “heavy lifting” (the intensive dose), using virtual tools only for longitudinal scaffolding. The digital format’s distinctive challenge is permeability. Unlike a residential retreat or in-person workshop, which physically remove participants from stressors, virtual delivery must demand ongoing attention and create psychological boundaries by design in order to retain the participants’ engagement. Structured schedules, small breakout groups, webcam expectations, group norms, and facilitated exercises can help, but they will generally compete with household demands and workplace interruptions. A search of the published, peer-reviewed literature yielded zero virtual synchronous programs that met the inclusion criteria for this review. Because digital interventions do not meet the inclusion criteria for this paper, they are incorporated here as adjacent conceptual literature to explore the impact of social density.

Several systematic reviews and meta-analyses have examined web- and app-based programs for stress, burnout, and related outcomes in working populations. Heber and colleagues, across 26 comparisons (*n* = 4226), found that web- and computer-based stress management produced a moderate effect on perceived stress (d = 0.43) and smaller effects on depression (d = 0.34) and anxiety (d = 0.32). Guided interventions (d = 0.64) outperformed unguided ones (d = 0.33) [85]. Wong and colleagues reviewed 17 RCTs for nurses during COVID-19 and found that diverse approaches could reduce anxiety, stress, and burnout, although evidence quality was uniformly low and heterogeneity was considerable [86]. Ladino and colleagues focused on Internet-based psychosocial interventions for burnout in healthcare workers and identified only four eligible RCTs; the pooled effect versus controls was not significant, underscoring the thin rigorous evidence for burnout specifically [87].

Most digital interventions are self-guided or minimally guided asynchronous programs: educational modules completed alone, at one’s own pace, sometimes with automated reminders. In the proposed framework, these programs are low in contextual containment, dose density, and social density. The market is dominated by self-paced apps for breathing, meditation, and CBT, often with limited evidence-based support [88]. “App fatigue” has also emerged. Rather than adding standalone tools whose proliferation has outpaced evidence of sustained use, the field may benefit from recommender systems that match users to existing services [89]. Even physicians who prescribe digital therapeutics report knowledge gaps and ambivalence, emphasizing that digital tools should complement, not replace, human connection [90].

Virtual synchronous programs attempt something different: they use live video to recreate the interpersonal dynamics and experiential engagement of in-person formats. Till and colleagues illustrated the importance of live social dynamics when they compared an onsite “Coping with Stress” seminar for medical students with an asynchronous online version and a no-intervention control [81]. The onsite seminar consisting of a 90-min small-group session with discussion, peer sharing, and facilitated reflection reduced emotional impairment (d = −0.18), exhaustion (d = −0.27), mental distance (d = −0.20), and stress (d = −0.33), and increased knowledge of help resources. The asynchronous version, in which students read materials and wrote reflective essays on their own time, changed neither stress nor burnout; it improved only knowledge. Live social interaction, not digital content alone, appeared to be the active ingredient.

When programs preserve live group process and experiential content, meaningful online effects appear possible. Moulton-Perkins and colleagues conducted the only systematic review focused specifically on synchronous group mindfulness delivered by videoconference. Across 10 studies, they found medium positive effects on mental health versus inactive controls (d = 0.44–0.71), with no significant difference from in-person delivery; however, no study was powered for noninferiority, and only three were of moderate-to-strong quality [91]. These findings suggest that when live video preserves group process and facilitator-led inquiry, outcomes may approximate in-person delivery, supporting social density rather than contextual containment as the operative dimension.

Additional studies point in the same direction. Wang and colleagues delivered a brief modified MBSR program by live videoconference to adults with anxiety during COVID-19 (six 60-min sessions over three weeks), and significantly reduced anxiety, depression, somatization, stress, and insomnia versus the waitlist. Benefits persisted at six months, did not differ significantly from a CBT active control, and attrition was low (6%) [92]. Al-Ghunaim and colleagues’ “Reboot-C”, a synchronous virtual coaching workshop for UK urology trainees delivered in two 2-h sessions, was associated with significant improvements in resilience, confidence, and burnout; participants experienced small-group breakout rooms as normalizing professional difficulty [93]. Yet virtual delivery showed a distinct vulnerability: some participants visibly disengaged or multitasked. Physical co-presence enforces baseline social accountability; virtual spaces remain permeable and allow participants to modulate engagement.

The largest controlled trial of a virtual group-based burnout intervention further supports the importance of live social process. A pilot study followed by a multisite RCT tested “Better Together Physician Coaching”, which combines live weekly videoconference coaching, anonymous written coaching, and self-paced modules for female physician trainees [94,95]. The pilot RCT (*n* = 101) found that the 6-month program significantly reduced emotional exhaustion and impostor syndrome and increased self-compassion versus the controls [94]. The multisite RCT across 26 institutions (*n* = 1017) replicated and extended these findings over 4 months, improving all three MBI subscales, moral injury, impostor syndrome, self-compassion, and flourishing, with a number needed to treat of 11 [95]. Because the content was distributed over months and participants remained embedded in their clinical environments, the recurring group-coaching calls functioned as facilitated peer support. Qualitative analysis identified connection and normalization of isolating challenges as central [95].

Where live videoconferencing replicates social dynamics without physical separation, virtual reality (VR) pursues the opposite strategy: it constructs an artificial environment that simulates removal from the workplace, creating a technologically mediated form of contextual containment [96,97]. By creating a sense of presence, VR may facilitate rapid physiological and emotional reset, and a growing body of work points to its role in stress reduction and burnout prevention [98,99]. Highly compressed VR sessions of 3 to 15 min can reduce emotional exhaustion, stress, and anxiety in fatigued healthcare workers, with high adherence because users need not leave the hospital [100,101]. VR also supports experiential multimodality through rich interactive stimuli such as nature navigation, 3D painting, and visually synchronized paced breathing, and appears more immersive and more effective at inducing relaxation than audio-only tools [102].

VR’s limits concern social density and scaffolding. It excels as individual escape but lacks the cooperative peer experience that builds group bonding. The abrupt return from an immersive sanctuary to a high-stress clinical environment can also be jarring [102]. VR and synchronous video therefore address complementary gaps: VR offers individual containment and multimodal engagement at the cost of social density, while live video preserves social density but struggles with containment and sensory engagement. Hybrid designs pairing brief VR “micro-doses” with live peer processing may capture both mechanisms, but this remains untested.

As with in-person intensives, the durability of virtual interventions remains uncertain and may be more fragile because participants never physically leave their environments. The boundary between “intervention time” and ordinary life is porous. Rather than compressing contact into a single block, virtual programs may be better suited to distributed sessions across weeks, creating gaps in which participants apply skills in real work settings. Qualitative evidence from an online mindfulness community of practice suggests that regular facilitated sharing rituals and low-pressure norms can help virtual groups develop mutual trust that partly substitutes for physical containment [103]. Participants described this as “connecting with ease”, and engagement that felt restorative rather than performative, echoing findings that digital interventions must feel supportive rather than administrative to sustain engagement [104].

Program design shapes uptake. Johnson and colleagues’ “EMPOWERed Classroom”, a two-session virtual workshop for postsecondary educators spaced 10 weeks apart, produced medium-to-large gains in mental health literacy and self-efficacy, highest after the second session [105]. Makowska-Tłomak and colleagues evaluated a blended intervention for employees facing digital-transformation stress. The program combined synchronous workshops with a self-guided platform, was grounded in the Job Demands–Resources model, and targeted self-efficacy. It significantly reduced disengagement in the active group while digital-transformation stress rose in the controls; dropout was 18%, far below the roughly 80% typical of self-guided programs [106]. This suggests that synchronous social components may sustain engagement when asynchronous content is included [106].

Digital stress creates a paradox: an intervention delivered digitally must not become another demand. Consistent with this concern, blended interventions that combined digital tools with human support achieved 72% adherence versus 31% for standalone apps [107]. Beliefs about benefit most strongly predicted acceptance, while Internet anxiety acted as a barrier that social influence and adequate facilitating conditions could buffer [108]. Overall, participants want interventions that are brief, contextually specific to their work, and peer-supported rather than solitary [104].

**Figure 2:**
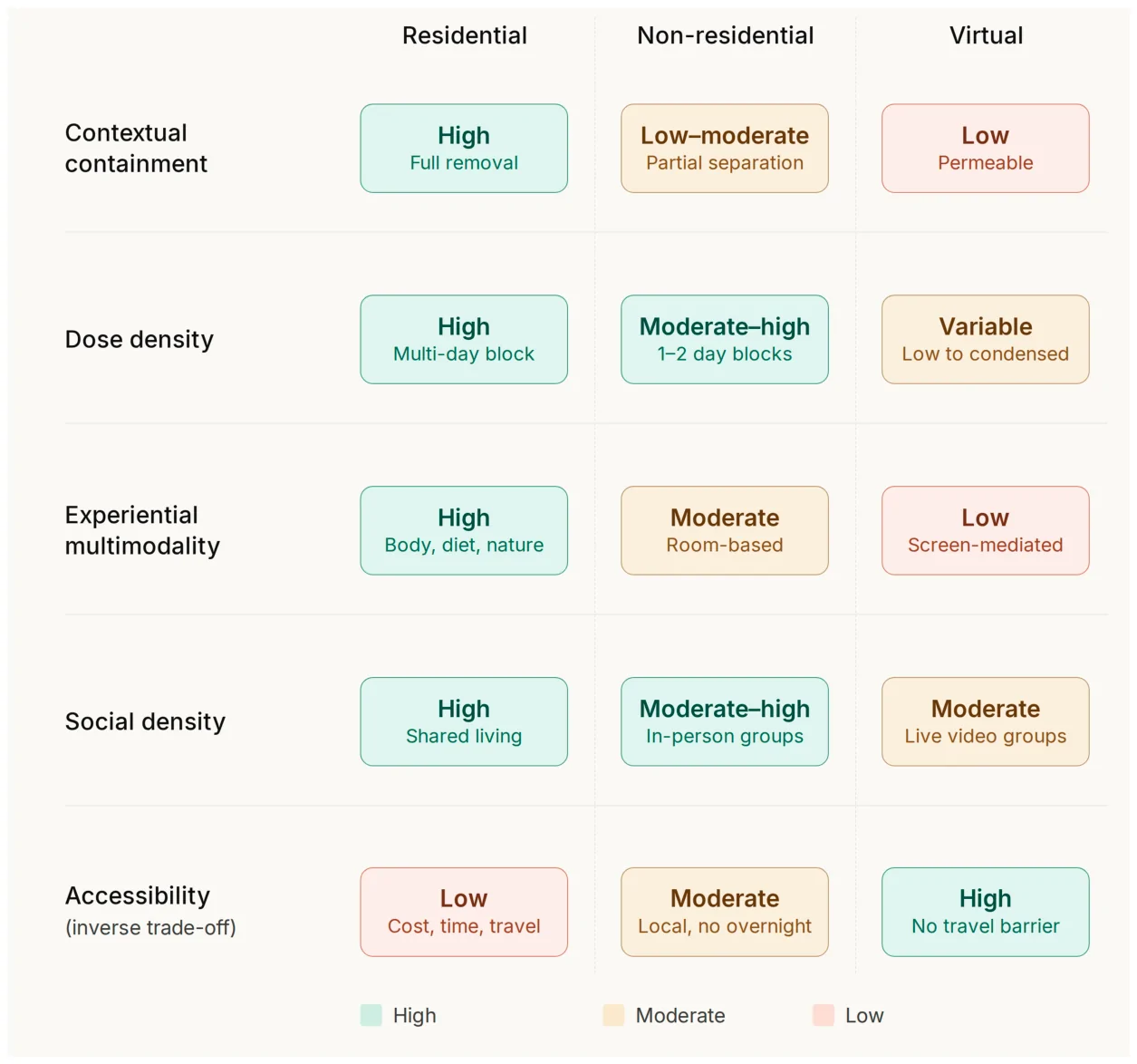
Working framework comparing residential, non-residential, and virtual delivery formats across the four immersion dimensions (contextual containment, dose density, experiential multimodality, social density) and accessibility. Ordinal ratings are anchored to the structural and temporal characteristics of the interventions reviewed in Section 4, Section 5 and Section 6.

No evidence table is provided for the virtual format because no identified study met this review’s full criteria for temporal compression. The evidence for virtual synchronous programs as immersive burnout interventions is thinner than the evidence for residential retreats or non-residential intensives: samples are generally small, controlled designs are rare, and no identified study directly compared synchronous virtual with in-person delivery of the same intensive content. The broader digital literature [85,86,87,88] shows modest effects, high attrition in unsupported formats, and human guidance or social connection as the ingredient most likely to distinguish effective from ineffective delivery. Adjacent work on intensive virtual spiritual retreats [109] and conceptual models of virtual retreat experiences in wellness tourism [110] is emerging but remains early. Whether high dose density in virtual formats can be adapted for burnout remains open. Figure 2 summarizes how the three formats compare across immersion dimensions and accessibility.

To operationalize this delivery-format framework (Figure 2), the ordinal ratings assigned to each format are anchored to specific structural characteristics and empirical examples drawn from the reviewed literature:

1. Contextual containment (removal from routine demands and everyday environments). Rated High when participants leave home and work overnight for the full program (e.g., isolated natural settings [38] or 6-day resort stays [39]). Rated Low–Moderate when participants leave work for the program but return home each night, so routine stressors partly reach into the recovery period (e.g., 6-h off-site workshops [58]). Rated Low when participants stay in their usual environment throughout, leaving the program open to interruption (e.g., visible multitasking during video sessions [93]).
2. Dose density (temporal compression into a single day or consecutive days). Rated High for multi-day blocks, typically several consecutive days with 5 to 10 h of daily programming (e.g., intensive 7-day retreats [47]). Rated Moderate–High for one or two days with typically 4 to 12 h of programming in total (e.g., a 12-h, 2-day training [49]). Rated Variable for virtual formats, which range from 60- to 120-min sessions spread over weeks, with little compression [92,94], to condensed multi-day formats now in registered trials (see Section The Emerging Horizon: Pipeline Virtual Intensives).
3. Experiential multimodality (practice-based use of multiple modalities rather than didactic content alone). Rated High when programs combine modalities, as is typical of residential retreats [37,38]. Rated Moderate when programs add room-based body or expressive exercises to cognitive content (e.g., progressive muscle relaxation and art therapy in non-residential intensives [54]). Rated Low when practice is limited to what can be guided through a screen, such as discussion, meditation, and breathing [91].
4. Social Density: Rated High when encompassing shared living, unstructured communal meals, and formal group vulnerability (e.g., overnight resident bonding [44]). Rated Moderate–High for in-person co-presence strictly limited to programmed hours (e.g., half-day workshops [66]). Rated Moderate–Low for synchronous live video, which successfully preserves facilitated peer support but lacks physical proximity and spontaneous informal interaction [91,95].
5. Accessibility (Inverse trade-off): Rated Low when requiring multi-day travel, lodging, and high cost. Rated Moderate for local 1- to 2-day non-residential workshops. Rated High for home-based virtual delivery with no travel barrier.

### The Emerging Horizon: Pipeline Virtual Intensives

While the published academic literature has historically avoided temporal compression in virtual formats, the commercial wellness market has increasingly adopted multi-day, synchronous virtual bootcamps and seminars. Academic evaluation of these formats is now emerging in trial registries. A search of ClinicalTrials.gov for synchronous virtual intensives targeting psychological well-being and occupational burnout identified an international registered trial (NCT04821037) that is currently evaluating a condensed virtual Acceptance and Commitment Therapy (ACT) intervention for frontline nurses, delivered via videoconferencing across two intensive half-day workshops. Stanford University’s Lasting Change Study (NCT05719129) evaluates the Date with Destiny seminar delivered virtually via videoconferencing. This matched cohort study represents a test of high dose density and social density delivered without contextual containment: participants engage in an intensive 6-day virtual seminar that is described as immersive and fully synchronous from their home environments. The study evaluates ~1400 participants using validated occupational metrics, including the Oldenburg Burnout Inventory and the Professional Fulfillment Index. These studies may provide preliminary evidence on the feasibility, effects, and durability of temporally compressed virtual delivery.

## 7. Discussion

### 7.1. Principal Findings and Interpretation

This review mapped emerging evidence on intensive interventions for burnout, stress, and occupational well-being across three delivery formats: residential retreats, non-residential in-person intensives, and virtual programs. We organized the evidence through an immersion framework with four dimensions: contextual containment, dose density, experiential multimodality, and social density. Across formats, studies frequently report short-term improvements and high participant satisfaction. However, a critical distinction must be drawn between indirect well-being evidence and direct occupational burnout evidence. While general stress markers, physiological biomarkers, and subjective well-being reliably improve across formats, direct evidence utilizing validated occupational burnout metrics is structurally thinner and markedly less consistent. Furthermore, the evidence is dominated by uncontrolled pre–post designs, small samples, healthcare-dominant and student-heavy populations, and short or absent follow-up. Controlled trials yield more conservative findings, and no identified study directly compares synchronous virtual and in-person delivery of the same intensive content. Overall, while the literature can describe what participants report experiencing, it cannot yet establish why these formats work, for whom, or for how long.

Residential retreats, as high-containment formats, appear to produce the clearest short-term shifts in stress-related and well-being outcomes [39,40,47]. Direct evidence for occupational burnout is thinner and less consistent, although the RISE program suggests that occupationally anchored retreats may improve proximal determinants such as self-compassion, sleep, and work engagement [29,30,35,36,45,71]. Non-residential intensives preserve dose and social density while weakening containment; they show a similar direction of effects but greater inconsistency and steeper fade-out [56,68]. Virtual synchronous programs offer greater reach but the published peer-reviewed evidence for their use as immersive burnout interventions remains the thinnest, though pipeline trials of temporally compressed virtual seminars are currently underway. Overall, this gradient requires caution. Axisa and colleagues’ RCT of a well-being workshop and Rollins and colleagues’ comparison of BREATHE with an active control found no significant between-group effects on burnout [51,57], suggesting that uncontrolled studies may overestimate intervention effects.

A persistent challenge is that the four proposed immersion dimensions covary across formats rather than vary independently. In this framework, residential retreats score high on all four; non-residential intensives preserve dose and social density while weakening containment; virtual programs may attenuate containment and multimodality together. This confounding-by-design means that larger or more durable effects in residential formats cannot be attributed to any single dimension. Wayne and Kaptchuk made a similar point in writing about tai chi as a multicomponent mind-body intervention: when therapeutic components are synergistic and inseparable, reductionist designs that isolate one active ingredient while controlling the rest may be neither feasible nor conceptually valid [111,112]. They also distinguished gross dosage (clock time) from effective dosage: the degree to which therapeutic processes are actually activated during contact. A 6-h in-person workshop with enforced presence and facilitated group work may deliver a qualitatively different dose than 6 h of virtual sessions with the same curriculum but frequent household or workplace interruptions [93].

Cross-format comparisons offer the best available leverage against this confound because they remove or attenuate one dimension while holding curriculum broadly constant. First, when contextual containment is removed by moving from retreat or in-person delivery to videoconference, synchronous group mindfulness can yield effects comparable to in-person delivery if facilitator-led group process is preserved, although no study was powered for equivalence [91]. Second, when temporal compression is removed by distributing coaching over months, the evidence can remain strong: the multisite Better Together trial (*n* = 1017) improved all three burnout dimensions while participants stayed embedded in ordinary environments [94,95]. Third, when live social interaction is removed and identical content is delivered asynchronously rather than synchronously, within-group improvements appear to fade: in a brief within-curriculum comparison, an onsite seminar reduced burnout and stress within groups, whereas its asynchronous counterpart improved only knowledge [81].

Contextual containment reliably produces large short-term shifts, but vacation-control designs suggest that much of this effect may reflect non-specific recovery rather than curriculum-specific benefit [39,40]. Social density therefore appears to be the best-supported candidate active ingredient. Temporal compression is the least tested dimension; its distinctive value beyond the social and recovery processes it bundles remains unproven.

Future studies should compare formats head-to-head, ideally by delivering the same manualized curriculum residentially, non-residentially in person, and virtually. They should measure all four immersion dimensions as continuous variables alongside validated outcomes. Multivariate approaches such as path analysis and structural equation modeling, as advocated by whole-systems researchers [111], may fit this problem better than between-group comparisons that treat format as a categorical variable.

### 7.2. Proposed Mechanisms of Change

**Figure 3:**
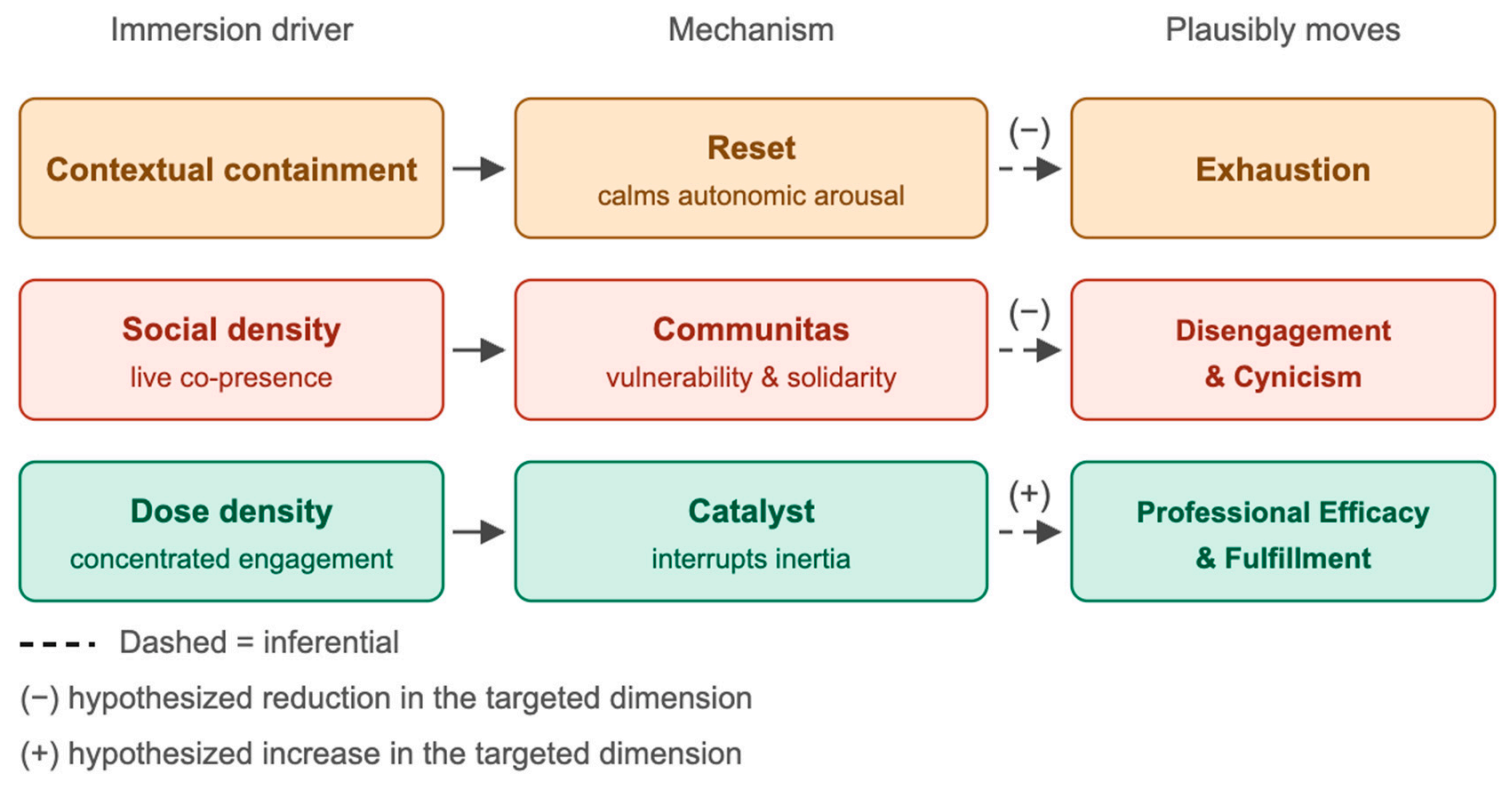
Three pathways of change linking immersion drivers to burnout dimensions. Contextual containment is hypothesized to act through a reset that lowers exhaustion; social density through communitas that lowers disengagement and cynicism; and dose density through a motivational catalyst that raises professional efficacy and fulfillment. Dashed arrows mark the inferential step from mechanism to durable, burnout-specific change, with (−) denoting a hypothesized reduction and (+) a hypothesized increase in the targeted dimension. These three pathways consolidate the four immersion dimensions of Figure 1.

The immersion dimensions described in this paper (Figure 1) can be consolidated into three pathways relevant to occupational burnout, each linking an intervention feature to the burnout dimension it may plausibly affect, as shown in Figure 3. The remainder of this section details each of these three proposed pathways. Contextual containment (removal from routine demands and everyday environments) may provide the reset needed to address exhaustion. Social density may produce communitas, an emotional bond that emerges when everyday social hierarchies, roles, and statuses dissolve, allowing people to connect as equals; this may loosen disengagement and cynicism. Dose density may act as a motivational catalyst for professional efficacy and fulfillment. No study in this review conducted mediation analyses of these pathways. The proposed pathways are therefore inferred and hypothetical, not demonstrated.

Exhaustion is the burnout dimension most often reported to improve. The contextual-containment-to-reset pathway rests on a view of burnout as failed recovery, not excessive load alone. Effort-recovery and allostatic-load models hold that strain becomes chronic when physiological activation is not reversed between episodes of demand [113,114]. Psychological detachment (mental disengagement from work during non-work time) is the recovery experience most consistently associated with lower exhaustion [115]. Contextual containment removes cues, communications, and role obligations that sustain activation. Mind-body practices in residential and non-residential intensives then occupy the space created by that removal [13,23,47]. Recovery gains may fade: vacation benefits erode rapidly on return to work [21], predicting the fade-out observed across intervention studies [59,68]. The effect may also be non-specific, because on-site vacation controls reproduce much of it [39,40], although meditation retreats outperformed ordinary vacations on fatigue and emotional well-being at follow-up [22].

The communitas pathway, which may improve disengagement and cynicism [34,49,54,58], draws on Turner’s account of liminality. Temporary removal from ordinary social structure suspends role and status distinctions and permits an intense, leveling form of solidarity [116]. Facilitated group process within such a bounded setting can help participants recognize that their distress is shared [117], producing group cohesion that the social identity approach to health treats as a psychological resource [118]. Cross-format evidence supports this pathway: removing live interaction while holding curriculum constant abolished measurable change [81]; preserving facilitator-led group process by video largely preserved effects [91]; and sustained group coaching produced effects without temporal compression [94,95]. Qualitative studies converge on shared vulnerability, normalization of distress, and peer connection as essential features of participation [31,62,71,93].

The limitation is that the group generating communitas often dissolves at the intervention’s exit. Unless a program is delivered to an intact social unit, the resource it creates may not exist in the environment to which participants return. Some of the most durable occupational results in this review come from a couples workshop that worked on an existing relationship and showed six-month effects on work exhaustion and interpersonal disengagement [31]. Whole-team and intact-cohort formats [53,61] should therefore be tested directly against stranger-group formats.

The dose-density-as-catalyst pathway proposes that concentrated engagement interrupts burnout-related motivational inertia and may improve professional efficacy and fulfillment. In the reviewed studies, professional fulfillment improved after a two-day retreat built on values clarification and action planning [27] and after a waitlist-controlled two-day couples intensive with booster sessions, where gains held at six months [31]. In a half-day workshop, personal accomplishment was unchanged at one month but significantly higher at six months, when stress gains had eroded [59]. Studies reporting no movement in this dimension [52,54,57,58,60] generally used shorter follow-up and the Maslach Burnout Inventory rather than an instrument built to detect the positive pole.

Within Job Demands–Resources theory, the motivational process predicts that depleted workers under-initiate the behaviors most likely to help them [2,3]. Intensive interventions demand attention, and temporal compression may circumvent the attrition that erodes multi-week programs [78,79]. They may generate enough commitment and self-efficacy [119] to launch practices that consolidate [25,26] and later improve professional efficacy and fulfillment. Participation may also prompt reflection and values clarification, leading to renewed vocational purpose or a career pivot. Such reappraisal is slow: transformative learning culminates in experimenting with new roles and reintegration [120], phases that unfold after the program and require longitudinal measurement. Finally, professional efficacy is tied more strongly to job resources than to demands [121], and immersive formats aimed at burnout and stress reduction are resource-additive. Two limits remain: no reviewed study measured motivation, self-efficacy, or work composition as an intermediate, and every program in which this dimension moved was also group-based or scaffolded, confounding dose density with social density.

The framework’s hypothetical claims can be tested in trials that measure recovery experiences, motivation, self-efficacy, group cohesion, values clarity, and work composition as candidate mediators alongside burnout dimensions. Such trials should use instruments that capture the positive pole separately and include follow-up long enough to detect delayed effects. They should also manipulate one dimension at a time: adding or withholding overnight containment from the same workshop, compressing or distributing the same curriculum, or delivering synchronous sessions with or without structured peer breakout groups.

### 7.3. Durability and Post-Program Scaffolding

Durability is the central unresolved problem for this intervention family. Awa and colleagues found that person-directed burnout interventions rarely persist beyond six months without refresher sessions [68], and the same pattern appears across the reviewed studies: stress improvements often erode while skill-related gains, such as mindfulness and self-compassion, prove more durable [59]. Burnout scores may even worsen after the intervention, once job demands reassert themselves [80]. Sustained change requires ongoing self-regulation, environmental support, and habit consolidation over weeks to months [25]. Immersive formats may therefore be better suited to initiation than maintenance unless they include follow-up support. Distributed booster sessions, digital nudges, peer communities of practice, and hybrid designs that pair an intensive block with spaced follow-up contacts warrant direct testing [106,108].

### 7.4. Limitations of the Evidence

Several methodological problems limit internal validity and generalizability. First, occupational coverage is uneven. Beyond healthcare professionals, trainees, health-professions students, and education professionals, the reviewed evidence includes a single study on senior public-sector managers [46], human services professionals [36,42,45], counselors, advocates, and lawyers [28], and behavioral health and social service staff [57,58], but each of these sectors is represented by one or a few small studies, usually uncontrolled. Blue-collar occupations and the self-employed are absent entirely, and corporate employees appear only in the adjacent virtual literature [106]. Whether findings extend to corporate employees, various public-sector occupations, blue-collar workers, or the self-employed remains unknown. Because burnout’s determinants and manifestations vary by context, this concentration constrains external validity. Second, participant satisfaction often diverges from demonstrated efficacy. Perceived benefit is nearly uniformly high, yet in the few controlled studies, it coexists with null or non-significant effects on validated measures [51,57]. High satisfaction may reflect the restorative experience of protected time away, social desirability when evaluating an employer-funded program, or genuine subjective benefit that current instruments miss. It cannot substitute for efficacy on validated outcomes.

### 7.5. Ethical, Practical, and Equity Considerations

The containment-accessibility trade-off in Figure 2 also has equity implications. Residential retreats require time away from work and family, travel, and often substantial personal or employer resources; workers in lower-paid, high-demand roles may therefore have the least access. Non-residential and virtual formats can reduce cost and travel barriers but introduce others, including digital fatigue and unequal access to protected space or reliable technology. The commercial uptake of burnout retreats and bootcamps heightens the need for independent evaluation, transparent reporting, and equity-focused implementation rather than promotional claims. Individual-level programs should be framed as adjuncts to organizational change, not substitutes for interventions in workload, staffing, role design, and leadership.

The scale of the commercial market sharpens these concerns. Residential retreats commonly charge $1000 to $5000 for a multi-day program, and luxury burnout-recovery retreats may charge far more [17]. The broader wellness-retreat market was valued at roughly $180–$225 billion in 2024 and is projected to exceed $350 billion by 2032, growing 7–10% annually [17]. Burnout-specific offerings, such as one-day “Burnout-Proof Bootcamps”, 7-day online resilience programs, 90-day “regenerative sabbatical” frameworks, and multi-week residential recovery packages, now market immersive formats directly to employers and individuals [122,123,124,125]. Many use confidently clinical language, such as “nervous-system repair”, “somatic release”, and “designed recovery systems” that outruns the published evidence [122,123,124,125].

Two risks follow. First, access is stratified: workers in lower-paid, high-demand roles are least able to reach the most intensive formats, while those who can afford luxury retreats may need them least. Second, immersive programs may become substitutes for organizational change rather than complements to it. The “re-entry shock” of returning from a transformative experience to an unchanged, high-demand workplace recurs in qualitative accounts [71] and fits the Job Demands–Resources model’s claim that individual resource gains cannot indefinitely offset structural demand overload [2]. No study in this review included an economic evaluation or reported the socioeconomic diversity of its sample, leaving it unknown whether these interventions reach the populations most affected by burnout.

### 7.6. Recommendations and Future Directions

Six priorities emerge. First, studies should compare formats head-to-head: the same manualized curriculum delivered residentially, non-residentially, and virtually, with the four immersion dimensions measured as continuous variables alongside validated burnout outcomes, consistent with whole-systems strategies advocated by Wayne and Kaptchuk [111,112]. Second, dismantling and dose-finding designs should isolate specific dimensions, such as a weekend workshop with versus without overnight residential containment, or synchronous virtual delivery with versus without structured peer breakout groups. Third, the field must move beyond healthcare workers and educators to public-sector, technology, the self-employed, and blue-collar workers, where the mechanisms, feasibility, and equity of immersive formats may differ.

Fourth, outcome measurement should be standardized: studies should include at least one validated multidimensional burnout instrument (MBI, OLBI, BAT, or PFI) at the baseline, post-intervention, and at three and six months, alongside work-functioning indicators such as absenteeism, turnover intention, and return-to-work status. Fifth, post-program scaffolding should be treated as a design variable; randomized comparisons of scaffolded and non-scaffolded versions of the same intensive should directly test the durability mechanisms. Sixth, economic evaluations are needed to assess cost-effectiveness and return on investment, given the cost of residential retreats and growing employer investment.

## 8. Conclusions

This review maps a growing but preliminary evidence base on residential retreats, non-residential intensives, and virtual synchronous programs for occupational burnout, stress, and well-being. Current studies support short-term gains in stress-related outcomes and proximal resources more consistently than durable change in validated burnout dimensions The strongest conclusion is not that immersive formats reliably reduce burnout, but that delivery format may itself be an active ingredient and should be studied explicitly. Evidence points most consistently to facilitated social connection as the best-supported intervention ingredient. Future work should test the delivery format as an active ingredient, extend follow-up and scaffolding, monitor safety and equity, and evaluate whether intensive formats complement the organizational changes required to reduce burnout.

## Figures and Tables

**Table 1:** Residential retreat studies (n = 22) evaluating burnout- and stress-related outcomes in adult working and occupationally relevant populations.

Study	Intervention(s)	Population	Intervention Dose/Length	Key Curriculum Components	Study Design & Comparator	Primary and Secondary Outcomes	Follow-Up Observation
**Direct Evidence (Validated Burnout Measures)**							
Bailey et al. (2023) [27]	“Intentional Practitioner Retreat”	Physicians (*n* = 13), APPs (*n* = 7), and resident (*n* = 1); family invited. *N* = 21 (assessment *n* = 12)	2 days; workshops + experiential activities	Burnout/stress psychoeducation; CBT and Intentional Change Theory; values clarification; mindful movement; “camaraderie conversations”; action planning.	Pre–post, within-subjects; no control. Wilcoxon signed ranks tests.	Significant decrease in Perceived Stress (*p* = 0.007) and increase in Professional Fulfillment (*p* = 0.041) Non-significant results for anxiety (GAD-7), Self-Reflection/Insight (SRIS), Mindful Self-Care (MSCS).	1-month planned; insufficient data (*n* = 3) for analysis.
Dutton et al. (2017) [28]	Joyful Heart Foundation (JHF) Holistic Healing Arts Retreat	Female counselors, advocates, and lawyers from community organizations who provide services to survivors of child abuse, domestic violence, and sexual assault (*N* = 18)	4 days (consisting of 2 half days and 3 full days of programming)	Grounded in a person-centered framework and holistic indigenous healing traditions. The retreat progressed through daily themes (Safety/Intention, Connection, Awareness, Expression, and Integration). Activities included psychoeducation on trauma and secondary trauma, body resourcing, present moment awareness, relaxation exercises, expressive movement and art, and group sharing. To ensure safety, participants were directed not to share past personal trauma narratives.	Open trial pilot study (uncontrolled pre- and post-retreat evaluation). No control group was utilized.	Participants demonstrated significant decreases in somatic and posttraumatic stress symptoms, perceived stress (PSS), depression, fatigue, insomnia, burnout (ProQOL), and secondary traumatic stress Participants showed significant increases in life satisfaction, appearance self-esteem, and the non-judging facet of mindfulness.	3- and 6- month follow up: The improvements achieved in somatic and PTSD symptoms, insomnia, perceived stress, depression, fatigue, burnout, and secondary traumatic stress were all maintained.
Dyer et al. (2020) [29]	RISE (Resilience, Integration, Self-awareness, Engagement) program	Education professionals *N* = 31 enrolled; RISE *n* = 9; control *n* = 21 100% female	3 days (~15 h total)	Yoga; mindfulness exercises; formal meditation; didactic sessions; experiential resilience activities.	Pragmatic controlled trial; waitlist control group.	Significant between-group differences favoring intervention (at post) (*p* < 0.05) for positive and negative affect, mindfulness, empowerment, self-compassion, work engagement. Non-significant differences in resilience and burnout (MBI-ES).	2-month follow-up; significant between-group improvements were sustained for stress, negative affect, mindfulness, empowerment, self-compassion, and sleep quality.
Dyer et al. (2021) [30]	RISE	Urban education professionals (primarily administrators). Analyzed sample *N* = 74	3 days (~15 h)	Yoga; breathwork; meditation; mindful communication; trauma-informed integration; daily home practice recommendation.	Single-arm pragmatic trial.	Post: Significant within-group improvements (*p* < 0.05) in stress, resilience, affect, mindfulness, empowerment, self-compassion, work engagement, burnout, and professional efficacy.	2-month follow-up; significant improvements sustained for resilience, positive and negative affect, mindfulness, empowerment, self-compassion, work engagement, professional efficacy, and exhaustion.
Gold et al. (2025) [31]	Couples-focused physician well-being weekend retreat + booster sessions	Physicians (*n* = 47 intervention group, *n* = 69 waitlisted control group participants)	2-day intensive workshop + 3 evening sessions	Positive psychology; gratitude; communication skills; relationship strengthening; discussion of physician culture traits.	Delayed-intervention waitlist-controlled cohort study. 6-month follow-up.	Significant improvements (*p* < 0.05) in Professional Fulfillment, burnout, self-valuation, and impact of work on personal relationships.	6 months; data were available for 38 intervention participants and 53 controls. All effects maintained.
O’Shea et al. (2022) [32]	Annual Emergency Medicine Residents Retreat	Emergency medicine residents (*N* = 60). Survey responders: *n* = 42 at baseline, *n* = 30 at 1-month follow-up.	2-day off-site retreat incorporating a 2.5-h mindfulness training	Mindfulness skills directly applicable “on shift” at work, mindful movement/standing body scan; discussion/inquiry; resources for continued practice, breathing exercises.	Uncontrolled intervention study (pre–post around retreat).	Significant decreases (*p* < 0.001) in stress (PSS) and emotional exhaustion as dimension of burnout. Non-significant results for mindfulness and other dimensions of burnout.	1 month post-retreat (T3): reductions in perceived stress and emotional exhaustion sustained at 1 month; no significant change in other factors.
Razzaque and Wood (2016) [33] †	Mindfulness-Based Professional Development (MBPD)	Psychiatrists, *N* = 26	2 days (48 h)	Foundational mindfulness meditation instruction; mindful walking/standing/eating; observation of thoughts/internal processes; linking mindfulness to clinical practice/empathy/therapeutic relationship; later teaching on mindfulness-based therapies/techniques for patients with emphasis on flexible models (e.g., ACT).	Mixed-method, uncontrolled pre–post with 1-week follow-up. Quantitative and qualitative analyses.	Significant improvements (*p* < 0.01) in burnout (MBI total score) and mindfulness. Non-significant results for self-compassion.	1 week post-retreat: mindfulness gains maintained; burnout reduced; working alliance increased; self-compassion not maintained at follow-up (overall NS).
Schroeder et al. (2016) [34]	Mindful Medicine Curriculum	Primary care physicians (family and internal medicine). Baseline *N* = 33 randomized (MMC *n* = 17; control *n* = 16).	13-h weekend training program (2 days) + two 2-h follow-up sessions at 2 and 4 weeks post-weekend	Modified MBSR including: mindfulness meditation, compassion skills, mindfulness-at-work training, secular framing.	Pilot randomized controlled trial (1:1 randomization to MMC vs. waitlist control). Patient satisfaction analyzed using aggregated 12-month baseline and 3-month post-intervention data.	Significant Group × Time effects (*p* < 0.05) for stress, mindfulness, emotional exhaustion, and depersonalization. Non-significant results for resilience, personal accomplishment, and compassion. Non-significant effects on patient-reported satisfaction.	3-month follow-up: All improvements maintained in MMC group. Majority (92%) reported ongoing formal and informal mindfulness practice at 3 months.
Trent et al. (2019a) [35]	RISE	Professional educators *N* = 57 consented; *n* = 44 baseline + post; *n* = 33 at 2-month follow-up.	3-day residential immersion at Kripalu. ~5 h/day structured sessions	Yoga, meditation, breathing techniques, mindfulness practices, sleep, resilience training.	Single-arm pre–post study. No control group.	Significant change (*p* < 0.01) for burnout exhaustion, burnout—professional efficacy, work engagement, perceived stress, resilience, affect, mindfulness, self-compassion, and satisfaction with-life.	At 2-months, maintained improvements in all outcomes except for stress and negative affect.
Trent et al. (2019b) [36]	RISE	Frontline professionals (education, health care, human services). *N* = 82 with *n* = 41 of waitlisted controls included.	5-day residential retreat	5-day residential immersion at Kripalu Center for Yoga & Health. ~5 h/day structured programming: ~2 h yoga instruction/practice, 90 min didactic, 90 min experiential sessions.	Controlled pragmatic trial.	Significant improvements in the intervention group (*p* < 0.01) for stress, resilience, affect, mindfulness, satisfaction with life and job satisfaction. Non-significant results for burnout dimensions and work engagement.	At 2-months, maintained improvements in stress, resilience and affect. The RISE group had new significant improvements compared to controls in empowerment and self-compassion.
**Indirect Evidence (Stress, General Well-Being, Biomarkers)**							
Blasche et al. (2021) [22]	(1) Residential meditation retreats; (2) Vacation-with-meditation; (3) Vacation-without-meditation.	Experienced meditators (M = 12.1 years practice); *N* = 120 completers.	Retreats: 14.5 days avg (33.8 h/week meditation); Vacations: 15 days avg	Shamata and Tonglen mindfulness meditation; informal mindfulness (walking, eating, chores).	Naturalistic observational design; 3 groups compared using GLM; Recovery experience (mastery, control, relaxation) as mediators.	Mindfulness was Significantly higher in both meditation groups vs. ordinary vacation at T2 and T3. Emotional Well-being: Higher in meditation groups vs. ordinary vacation at T3 (d = 0.70–0.81). Fatigue (FAS): Lower in meditation groups vs. ordinary vacation at T3 (d = 0.62–0.80).	10 weeks; meditation (retreat or vacation-based) sustained well-being and reduced fatigue, while ordinary vacation effects faded.
Cohen et al. (2017) [37] †	Holistic residential wellness retreat	Wellness tourists (*N* = 37); 65% female; mean age 48.7 years; mostly omnivorous.	1 week (7 days); structured immersive experience	Organic plant-based diet; physical activities (vigorous and restorative); educational wellness talks; “dreamtime” (rest and spa treatments); digital detox; circadian alignment.	Longitudinal observational study; No control group; Paired-samples *t*-tests.	Psychological/Health: Significant improvements in wellness inventory, mood states, insomnia, depression, anxiety, and self-efficacy. Urinary pesticide metabolites and anthropometrics were also measured.	6 weeks; improvements in wellness, mood, self-efficacy; depression, anxiety, and insomnia and scores drifted back toward baseline.
Djernis et al. (2021) [38]	(1) Indoor mindfulness retreat; (2) Natural outdoor retreat; (3) Passive control.	Danish university students with elevated stress (PSS ≥16); Novice meditators. *N* = 60 (*n* = 20 indoor, *n* = 22 nature, *n* = 18 control).	5 days; intensive residential format	Modified 8-week MBSR curriculum; morning/evening sitting meditation; yoga; silent formal and informal mindful walks; emphasis on self-compassion.	Pilot RCT; 3-armed parallel design. Mixed linear models.	Self-Compassion (SCS-SF): Significant improvement for both intervention groups at follow-up (g = 0.49–0.54). Stress (PSS): Non-significant reduction, but clinically relevant effect post-intervention for indoor (g = 0.56). Mindfulness: Significant post-treatment improvement in behavioural task (BCT) for both groups (g = 0.90–0.94).	3 months; sustained improvements in self-compassion and self-reported mindfulness for both groups.
Epel et al. (2016) [39] †	(1) Mantra meditation retreat; (2) On-site vacation control.	Healthy women (*N* = 94); randomized to novice meditators (*n* = 33) or vacation control (*n* = 31), plus a non-randomized cohort of experienced meditators (*n* = 30).	6 days; residential	Mantra-based meditation; yoga; self-reflection; group lectures; Ayurvedic-style diet.	Randomized controlled trial (for novices) + non-randomized experienced cohort; vacation control group.	Well-being: Significant improvements across all groups post-stay (*p* < 0.05). Biological “Reset”: Significant transcriptome-wide changes in 1792 genes related to stress and immune response across all groups (*p* < 0.05).	10 months; novice retreat group showed significantly more sustained improvements in stress and depression than the vacation-only group (*p* < 0.05).
Gardi et al. (2022) [40]	Residential Mindfulness Retreat (RMR)	Healthy adults (*N* = 95 randomized; *n* = 46 RMR, *n* = 44 control analyzed).	3 days	Vipassana/Zen-based; ~10 h/day of alternating sitting/walking meditation; “noble silence”.	RCT with active residential vacation control.	Significant decreases (*p* < 0.001) in perceived stress, cortisol levels, anxiety, IL-6 and IL-8; Significant increase in awareness and IL-10 (*p* < 0.001) Within-group (Vacation): Sig. lower PSS (*p* = 0.002).	No long-term follow-up; pre- and post-intervention assessments only.
Montero-Marin et al. (2016) [41] †	Vipassana meditation retreat (vs. matched experienced meditator controls continuing usual daily practice)	Experienced meditators: Intervention group: *n* = 19. Control group: *n* = 19 (matched experienced meditators).	1-month residential retreat (8–9 h/day)	Vipassana practice (focused attention + open monitoring); Mostly unguided practice; Group meditation (weeks 1 & 4); solitary practice (weeks 2 & 3); Silence, isolation from external contact; vegetarian.	Controlled, non-randomized, pre–post intervention trial; Matched experienced meditator control group.	Significant retreat vs. control differences (ANCOVA) in non-attachment, positive affect, cooperativeness, reward dependence. Non-significant results for negative affect, life satisfaction, harm avoidance, and novelty-seeking.	No follow-up; pre–post only (1 month).
Pidgeon et al. (2014) [42] †	Mindfulness with Metta Training Program: 2.5-day residential retreat + two 4-h booster sessions	Human services professionals recruited from a not-for-profit community organisation *N* = 44; 91% female.	2.5 days (weekend). Booster sessions: 4 h each, delivered over ~12 weeks (at 1 month and 4 months)	Mindfulness + loving-kindness experiential exercises; cognitive therapy model of disturbing emotions; skills to notice body sensations/thoughts; mindful awareness tools to respond vs. react.	Pilot randomized trial: MMTP retreat group vs. no-intervention control. Measures at baseline (pre-retreat) and immediate post-retreat for both groups; retreat group also assessed at 1-month and 4-month follow-up.	Non-significant between-group results for resilience, mindfulness, and self-compassion; Within retreat group significant results (*p* < 0.05) for resilience, mindfulness, and self-compassion.	Follow-up at 1 month and 4 months (aligned with boosters). Improvements observed at 1 and 4 months for mindfulness and self-compassion; resilience improvement observed at 4 months.
Taren et al. (2015) [43] †	Health Enhancement through Mindfulness vs. Health Enhancement through Relaxation (active control)	Community adults. RCT sample: *N* = 35 randomized (HEM *n* = 18; HER *n* = 17).	Residential 3-day retreat (condensed adaptation of 8-week MBSR).	HEM (mindfulness): body scan awareness, meditation, mindful eating and movement, group discussion, self-care and stress relief emphasis. HER: walking, stretching didactics; emphasis on relaxation; group support.	Single-blind randomized controlled trial (HEM vs. HER).	Right amygdala-subgenual ACC rsFC significantly decreased in HEM vs. HER; Higher perceived stress associated with greater bilateral amygdala-sgACC rsFC.	4 months: No significant group differences in hair cortisone or cortisol at 4 months.
Taya et al. (2024) [44]	Annual wellness-focused off-campus weekend retreat for radiology residents	Radiology residents at a single medical center; 78 retreat attendances, respondents *N* = 75.	Two-night weekend retreat	Resident-designed programming aimed at promoting well-being and social cohesion. Structured elements common across years: group exercise, meditation, nutritional wellness, team-building, outdoor activities, unstructured free time	Single-arm pre–post survey study (cross-sectional within each retreat year). Paired *t*-tests comparing pre- vs. post-retreat scores. Linear regression for effect modification by year and training level. No control group.	Significant results (*p* < 0.01) for perceived stress, resilience, and cohesion.	No longitudinal follow-up beyond immediate post-retreat surveys.
Trent et al. (2018) [45]	RISE	Frontline professionals (education, health care, human services). Recruited *n* = 75; post-completers *n* = 55.	5-day residential retreat	5-day residential immersion at Kripalu Center for Yoga & Health. ~5 h/day structured programming: ~2 h yoga instruction/practice, 90 min didactic, 90 min experiential sessions.	Single-arm pre–post pilot study (no control group). Paired samples *t*-tests across baseline, post, 2-month follow-up.	Significant change (*p* < 0.01) for perceived stress, resilience, affect, mindfulness, and empowerment, and sleep quality along with health behaviors. Non-significant results for compassion.	2-month follow-up: (*n* = 40): maintained improvements in stress, resilience, affect, mindfulness, empowerment, fruit/vegetable intake, and sleep quality vs. baseline (all *p* < 0.01). Exercise gains not sustained.
Veach et al. (2003) [46]	Stress workshop retreat	US governmental senior managers (*N* = 95 attended; *n* = 83 completed baseline and follow-up assessments).	3.5 days (total of 35 h of programming)	Didactic and experiential learning mix: Mind-body exercises (yoga, Tai Chi, or walk/run) followed by seminars on stress, coping skills, and lifestyle medicine using the Stress and Coping Inventory framework; Adventure-based learning for team building; One-on-one confidential counseling sessions, relaxation training.	Uncontrolled pre- and post-intervention study.	Significant improvements (*p* < 0.05) were found in overall Global Balance scores, psychological symptoms, depression (Zung scale), Health Habits, Social Support, Responses to Stress (both positive and negative), and Life Satisfaction	10 months post-workshop. The improvements in Global Balance scores, psychological symptoms, depression, and coping scales were sustained at 10 months.
Zangri et al. (2024) [47]	Mindfulness-Based Stress Reduction (MBSR) retreat delivered in intensive residential format	Healthy adults recruited from the community, *n* = 49, randomized: MBSR-first: *n* = 25; CCT-first: *n* = 24.	7-day residential retreat (condensed MBSR format). Daily practice of ~9 h/day	MBSR (3-day intensive format): Sitting meditation, Body awareness, Mindful movement, Inquiry and discussion. CCT (3-day intensive format): Mindfulness foundation, compassion practices, Emotion-focused contemplative exercises.	Randomized sequential crossover design.	Significant main effect for emotion regulation, self-compassion, compassion, and mindfulness. No significant Order × Time interactions for stress, anxiety, or depression. Side effects reported: emotional sensitivity, sleep difficulties, headaches, sensitive hearing.	3-month follow-up maintained emotion regulation improvements, self-compassion, mindfulness of mental events, fear of compassion reduction

**Note**: The studies provide either direct evidence evaluating occupational burnout using validated instruments [27,28,29,30,31,32,33,34,35,36] or indirect evidence evaluating stress, general well-being, or biomarkers [22,37,38,39,40,41,42,43,44,45,46,47]. **Abbreviations**: ACC, anterior cingulate cortex; ACT, Acceptance and Commitment Therapy; ANCOVA, analysis of covariance; APPs, advanced practice providers; BCT, Breath Counting Task; CBT, cognitive behavioral therapy; CCT, Compassion Cultivation Training; FAS, Fatigue Assessment Scale; GAD-7, Generalized Anxiety Disorder 7-item scale; GLM, general linear model; HEM, Health Enhancement through Mindfulness; HER, Health Enhancement through Relaxation; IL-6, IL-8, IL-10, interleukin-6, -8, -10 (inflammatory cytokines); JHF, Joyful Heart Foundation; MBI, Maslach Burnout Inventory; MBI-ES, Maslach Burnout Inventory–Educators Survey; MBPD, Mindfulness-Based Professional Development; MBSR, Mindfulness-Based Stress Reduction; MMC, Mindful Medicine Curriculum; MMTP, Mindfulness Meditation Training Program; MSCS, Mindful Self-Care Scale; N/n, sample size; ProQOL, Professional Quality of Life scale; PSS, Perceived Stress Scale; PTSD, post-traumatic stress disorder; RCT, randomized controlled trial; RISE, Resilience, Integration, Self-awareness, Engagement program; RMR, Residential Mindfulness Retreat; rsFC, resting-state functional connectivity; SCS, Self-Compassion Scale; SCS-SF, Self-Compassion Scale–Short Form; sgACC, subgenual anterior cingulate cortex; SRIS, Self-Reflection and Insight Scale. † **Overlap Note**: Primary data from Epel et al. [39], Taren et al. [43], Pidgeon et al. [42], and Cohen et al. [37] were previously included in the broad wellness retreat review by Naidoo et al. [20]. Cohen et al. [37], Montero-Marin et al. [41], and Razzaque and Wood [33] were included in the meta-analysis by McClintock et al. [48]. These studies are extracted individually here to evaluate occupational burnout and stress parameters through our structural delivery framework.

**Table 2:** Non-residential in-person intensive studies (*n* = 18) evaluating burnout- and stress-related outcomes.

Study	Intervention(s)	Population	Intervention Dose/Length	Key Curriculum Components	Study Design & Comparator	Primary and Secondary Outcomes	Follow-Up Observation
**Direct Evidence (Validated Burnout Measures)**							
Alenezi et al. (2019) [49]	Burnout prevention program	Mental health nurses in Saudi Arabia (*N* = 296 allocated; *n* = 255 completed: *n* = 130 intervention, *n* = 125 control).	12 h total (delivered over 2 days, 6 h per day)	Introduction to burnout and prevention principles; self-care activities (deep breathing, progressive muscle relaxation); stress reduction management; social skills training; and communication skills. The curriculum was culturally adapted for Arabic/Saudi customs.	Quasi-experimental, non-equivalent pre-test and post-test design. The comparator was a control group of nurses at a different hospital who did not receive the intervention.	At 1 month, the intervention group showed a significant decrease in emotional exhaustion and depersonalization and a significant increase in personal accomplishment (MBI, *p* < 0.001).	Evaluated at 1, 3, and 6 months. At 6 months, while scores declined from their 1-month peak, all three burnout (MBI) subscales remained improved compared to the baseline and to the control group.
Amirianfar et al. (2025) [50]	One day wellness retreat	Physical Medicine & Rehabilitation trainees (*N* = 26; 24 residents and 2 fellows).	9 h; 8:00 am to 5:00 pm	Morning (Indoor): lectures and discussion on wellness, burnout, and leadership; self-reflection exercises to develop goals; a 1-h yoga class; and 20 min of dog therapy. Afternoon (Outdoor): 4 h of team-building activities and bonding at a local golf range	Single-center, quasi-experimental pre–post design. No control group.	Burnout measured using the Copenhagen Burnout Inventory, which includes subscales for total, personal, work-related, and client-related burnout. The study found non-statistically significant improvements across all burnout scores following the retreat.	Assessed at 3 weeks and 3 months. The reductions in burnout were not statistically significant at follow-up, although the authors observed a trend of declining burnout that was most pronounced at 3 months.
Axisa et al. (2019) [51]	Well-being Workshop	Australian basic physician trainees in Adult Internal Medicine and Pediatrics (*N* = 46; *n* = 23 intervention, *n* = 23 control).	4.5 h (single half-day session)	Strategies for well-being and stress management; tailored case studies; group work activities; mindfulness; giving/receiving feedback; and dealing with conflict.	Randomized-controlled trial. The comparator was a control group that did not attend the workshop.	The workshop did not yield any significant immediate or at-1-month improvements across depression, anxiety, stress, traumatic stress, burnout, compassion satisfaction, or alcohol use outcomes.	At 6 months, there was a small reduction in alcohol use, depression, and burnout in the intervention group, but none of these changes reached statistical significance.
Durant et al. (2025) [52]	Mindful self-compassion workshop incorporating group sandtray exercises	Undergraduate nursing students (*N* = 133 total; *n* = 68 participants, *n* = 65 control subjects).	4 h	Group sandtray builds; foundational education on the “Window of Tolerance”, self-compassion; mindfulness (grounding, breath, reflection, movement); and trauma-informed care principles	Mixed methods pre–post quasi-experimental design. The comparator was a convenience sample of nursing students who did not undergo the intervention.	In the multivariate difference-in-differences analysis, the intervention group experienced 71% reduced odds of feeling burned out from their studies once a week or more compared to the controls (MBI-S instrument). No significant change in the specific domain total scores for exhaustion, cynicism, and professional efficacy compared with control subjects after controlling for lifestyle factors.	2 months post-intervention with attrition at approx. 50%; intervention participants experienced 71% reduced odds of feeling burned out from their studies once a week or more compared to the control group.
Hill et al. (2010) [53] †	Stress and Burn-out Training	Multidisciplinary staff on an in-patient alcohol ward (*N* = 19).	2 days (off-site)	Day 1 focused on managing stress at the individual, team, and organizational levels. Day 2 focused on understanding the causes and consequences of aggression, covering identification of antecedents, comprehensive risk assessment, and implementing effective risk management	Uncontrolled pre- and post-intervention evaluation. No control group	A statistically significant increase in feelings of personal accomplishment (MBI, *p* < 0.005). Non-significant results for emotional exhaustion and depersonalization dimensions of burnout (MBI)	None beyond the 1 month assessment.
Kravits et al. (2010) [54]	“Self-Care Strategies for Nurses” psycho-educational program	Nurses (including new graduates, incumbent cancer center staff, and community nurses) and a small number of non-RN health providers (*N* = 248).	6-h single session	Psycho-education on the stress response, the relaxation response, and both general and nursing-specific coping patterns. The curriculum utilized art therapy extensively to explore coping options, alongside guided deep breathing, grounding exercises, guided imagery, positive intention practice, progressive muscle relaxation, and the creation of a personalized wellness plan.	Uncontrolled pre- and post-intervention evaluation; no control group.	Immediately post-course, participants showed a statistically significant decrease in emotional exhaustion, and depersonalization dimensions of burnout (MBI-HSS, *p* < 0.0005).	None.
Long Franco & Christie (2021) [55]	One-day workshop adaptation of the Self-Compassion for Healthcare Community Program	Pediatric nurses (both clinical and non-clinical) at an urban pediatric hospital. *N* = 48 analyzed (*n* = 22 in the intervention group; *n* = 26 in the comparison group).	1-day format with six active hours total	Writing activities (e.g., how to treat a friend vs. oneself, writing a compassionate note, core values); short facilitator-led practices (finding a supportive gesture, moments of mindfulness, self-compassion breaks); a mid-day group reflection exercise; and access to coloring supplies and small toys as stress-diversion tools.	Quasi-experimental, prospective design utilizing pre- and post-intervention surveys; The comparator was a non-randomized control group of peer nurses who did not attend the training.	Compared to controls, the intervention group exhibited increases from pre- to post-intervention in self-compassion, mindfulness, and compassion satisfaction, as well as significant decreases in burnout (ProQOL), anxiety, and stress (DASS) (*p* < 0.05). No significant group-by-time interaction for secondary traumatic stress, depression, resiliency-activation, or job engagement.	At 3 months, The significant improvements in self-compassion, mindfulness, compassion satisfaction, burnout, anxiety, and stress were all maintained.
Morrow et al. (2022) [56]	Four distinct mindfulness-based interventions: Standard Mindfulness Based Stress Reduction (MBSR), Everyday Mindfulness (EM), Mindfulness in Medicine (MiM), and Restoring Balance (RB)	Healthcare professionals at an academic medical center and affiliated cancer hospital. *N* = 86 respondents across pre- and post-surveys.	Varied by program. MBSR: 2.5 h per week for 9 weeks + a 7-h retreat day (~29.5 h total); EM: 2 h per week for 4 weeks (8 h total); MiM & RB: 8 h (1-day retreats)	Sitting/walking meditations, body scans, restorative yoga, mindful communication, and didactic discussions on stress, burnout, and compassion fatigue; narrative medicine, appreciative inquiry, large-group “Big Mind” processes, and written self-care planning	Mixed-methods, uncontrolled pre–post survey design; no control group.	Post-survey respondents demonstrated significantly reduced burnout (*p* = 0.031, single item) and lower perceived stress (PSS, *p* = 0.007) compared to pre-survey respondents. There were no statistically significant differences in outcomes based on which of the four classes the participants took.	None.
Rollins et al. (2016) [57]	BREATHE (Burnout Reduction: Enhanced Awareness, Tools, Hand-outs, and Education) workshop	Behavioral health providers across three U.S. Department of Veterans Affairs (VA) facilities and two community social service agencies (*N* = 145 randomized; *n* = 76 BREATHE, *n* = 69 control).	1 full day-long workshop	Core content includes cognitive-behavioral techniques (e.g., deep breathing, cognitive restructuring), contemplative practices (e.g., mindfulness meditation, imagery, yoga), social support building, physical wellness education (sleep hygiene and exercise), and time management. Participants also used a workbook to create a self-care plan	Randomized controlled trial; The active control condition was a parallel daylong workshop focused on person-centered treatment planning (minimal overlap with the BREATHE curriculum)	Within-group small improvements in cynicism at six weeks, and in emotional exhaustion (*p* < 0.05) No significant improvements over time for reduced personal accomplishment, job satisfaction, turnover intentions, work absences, or work-life balance No significant differences between the intervention and control groups across any outcomes	At 6 weeks and 6 months post-intervention; participants showed small improvements in emotional exhaustion and positive views or expectations of clients (*p* < 0.05) No significant differences for reduced personal accomplishment, job satisfaction, turnover intentions, work absences, and work-life balance
Salyers et al. (2011) [58]	BREATHE (Burnout Reduction: Enhanced Awareness, Tools, Handouts, and Education)	Community mental health and substance abuse providers, including direct-care, administrative, and support staff (*N* = 84 attended; *n* = 74 completed follow-up).	6 h (single day session)	Burnout functional analysis and relapse prevention framework; skill-building in six areas: contemplative (mindfulness), social (setting limits), physical (body scan), cognitive-philosophical (cognitive restructuring/values clarification), imagery, and general self-care. Participants created a personalized burnout prevention plan.	Uncontrolled pre–post quasi-experimental design; no control group.	At 6 weeks post-training, the intervention group demonstrated significant decreases in emotional exhaustion and depersonalization (*p* < 0.01), and a significant increase in consumer optimism (*p* < 0.05). no significant changes in personal accomplishment, job satisfaction, or intention to leave.	No further follow-up beyond 6 weeks.
Sarazine et al. (2021) [59]	Mindfulness Workshop	Nurses at a Midwest academic medical center (*N* = 52 completed baseline; *n* = 20 completed 6-month follow-up).	4 h (single half-day session)	Mindfulness-based training and skills development.	Uncontrolled pre- and post-intervention survey design; no control group.	At 1 month post-intervention, significant decrease in perceived stress (PSS) and emotional exhaustion (MBI, *p* < 0.05). No significant effect on mindfulness skills, personal accomplishment and depersonalization.	At 6 months, significant findings included increased perceptions of mindfulness and personal accomplishment and a decrease in emotional exhaustion (*p* < 0.05). Improvements in stress were no longer significant.
Żołnierczyk-Zreda (2005) [60] †	Stress management workshop grounded in Karasek’s Job-Stress model	Teachers (*N* = 58 analyzed; *n* = 29 in the experimental group, *n* = 29 in the no-intervention control group).	2 days (6 h per day)	Activities included time management, cognitive restructuring (e.g., addressing perfectionism), relaxation/detraction exercises, assertiveness training, skills for handling disruptive student behaviors (like overt/passive aggression), and identifying positive meanings in their work.	Randomized quasi-experimental design utilizing pre-intervention (T1) and post-intervention (T2) assessments; the comparator was a no-intervention control group.	The intervention group demonstrated significant decreases in emotional exhaustion, perceived workload (intellectual demands, overload, and role conflict), behavioral job control, and the intensity of somatic complaints compared to the control group. No significant intervention effects on the MBI dimensions of personal accomplishment or depersonalization, perceived social support and the frequency of somatic complaints.	The primary post-treatment assessment was conducted at 1-month post intervention; no further follow up.
**Indirect Evidence (Stress, General Well-Being, Biomarkers)**							
Haber et al. (2020) [61]	Residency wellness retreat	Radiology residents (*N* = 37 participated; *n* = 33 completed the questionnaire)	1 day (8.5 h)	Teambuilding and bonding including outdoors; breakout session with brainstorming solutions for reducing burnout, improving teaching, and fostering camaraderie; confidential, guided group reflection.	Uncontrolled, post-intervention anonymous survey evaluated as a quality improvement initiative; no control group.	97% of respondents indicated the retreat would improve their residency experience, and 82% anticipated it would improve their personal wellness.	None.
Lucre et al. (2026) [62]	One-day “Compassionate Mind Training (CMT) for ALL”	Mental health staff across disciplines, including Allied Health Professionals, nursing, management, medics, and admin/clerical staff (*N* = 455 completed evaluation forms).	1 day (approximately 7 h)	Psychoeducation, the “Three Circles” model of emotional regulation (threat, drive, and soothe systems), exploring fears, blocks, and resistances to compassion. The curriculum utilizes play, movement, and experiential activities, breathing/mindfulness practices, and group reflections.	Qualitative service evaluation using thematic analysis of post-workshop feedback forms; no control group.	Participants reported a new comprehension of compassion and feelings of guilt associated with prioritizing self-compassion; they reported finding new techniques to soothe themselves; high perceived workshop value, and appreciation for integrative teaching methods.	None for this evaluation.
Manocha et al. (2009) [63]	“Mental silence” (Sahaja yoga) meditation seminar and subsequent home practice	General Practitioners (*N* = 299 attended; *n* = 111 completed both pre- and post-intervention assessments).	A single-session of 3 to 5 h, followed by an optional 2 weeks of twice-daily home practice.	A 1-h lecture on GP well-being; a 45-min lecture on meditation and its benefits; a large-group guided meditation; and small-group facilitated meditation skills practice; Participants also received a home practice kit.	Uncontrolled pre- and post-intervention evaluation; no control group.	Significant reductions in psychological distress, with the mean score dropping from 17.2 to 14.7 (*p* < 0.001); 25.1% of participants categorized as “at risk” shifted to the “low risk” category. Over 93% of participants reported improvements in mental silence, calm, and stress/tension on the VAS.	2 weeks post-seminar; the self-rated experience of mental silence improved significantly by 40% from day 1 to day 14; reporting a higher level of mental silence was correlated with experiencing lower distress.
Martin et al. (2021) [64]	Mindfulness-based intervention (MBI) workshop	Health professions students across nursing, athletic training/kinesiology, nurse anesthesia, and nutrition (*N* = 39 at baseline; *n* = 27 completed follow-up).	A single, brief workshop	Didactic content covering mindful awareness, mindfulness in daily life, the physiology of stress/relaxation, and self-compassion. Included brief guided practices such as mindful breathing, compassionate breathing, mindful eating (utilizing a sensory experience with a chocolate square), and reflective journaling.	Uncontrolled pre- and post-intervention survey design, no control group.	At 2 months post-intervention, participants reported a statistically significant increase in self-compassion (*p* < 0.05) and a statistically significant decrease in perceived stress (*p* < 0.01) compared to the baseline.	None beyond the 2-month assessment.
Poulsen et al. (2015) [65]	Interventional Recovery Workshop	Radiation therapists and oncology nurses (*N* = 70 evaluable; *n* = 30 intervention, *n* = 40 control).	1 day (single day session)	Evidence-based recovery techniques targeting psychological detachment, relaxation, mastery, and control; a module on social support during goal-setting utilizing peer mentoring, and mental contrasting with implementation intentions.	Equivalent, randomized comparison pretest-posttest design. The comparator was a control group that received the written educational material only.	At 6 weeks post-workshop, the intervention group showed greater mean changes compared to the control group in total recovery experiences (REQ), satisfaction with self-care, and perceived sleep quality (*p* < 0.05).	None beyond 6 weeks.
Roy et al. (2019) [66]	Introduction to well-being” interprofessional workshop	First-year medical and nursing students (*N* = 251 total; 210 medical students and 41 nursing students).	A single afternoon workshop where small groups (12–15) rotated through 3 concurrent sessions)	Self-care: breathing exercises, guided self-compassion meditation, discussions on the physical manifestations of stress and asking for help; Empathy: Utilizing multimedia (short films) to illustrate patient perspectives, role-play for communication.	Post-intervention qualitative evaluation using realist thematic analysis of written feedback forms; no control group or pre-intervention baseline.	Participants reported that the interprofessional sharing of experiences and the practical tools (like breathing techniques) were highly beneficial; conclusion that simple acts of care toward oneself and others are essential for maintaining well-being.	None.

**Note**: These studies can be distinguished into those evaluating direct occupational burnout outcomes [49,50,51,52,53,54,55,56,57,58,59,60] and those evaluating indirect stress, recovery, or well-being markers [61,62,63,64,65,66]. **Abbreviations**: BREATHE, Burnout Reduction: Enhanced Awareness, Tools, Handouts, and Education; CBI, Copenhagen Burnout Inventory; CMT, Compassionate Mind Training; DASS-21, Depression, Anxiety and Stress Scale–21 item; EM, Everyday Mindfulness (program arm); GP, general practitioner; MBI, Maslach Burnout Inventory; MBI-S, Maslach Burnout Inventory–Student Survey; MBSR, Mindfulness-Based Stress Reduction; MiM, Mindfulness in Medicine (program arm); N/n, sample size; ProQOL, Professional Quality of Life scale; PSS, Perceived Stress Scale; RB, Restoring Balance (program arm); RCT, randomized controlled trial; REQ, Recovery Experience Questionnaire; VA, U.S. Department of Veterans Affairs; VAS, visual analogue scale. † **Overlap Note**: Primary data from Hill et al. [53] and Żołnierczyk-Zreda [60] were previously included in the broader systematic reviews by Pijpker et al. [67] and Awa et al. [68], respectively. They are extracted individually here to analyze their specific compressed delivery formats.

## Data Availability

No new data were created or analyzed in this study. Data sharing is not applicable to this article.

## Supplementary Materials

The following supporting information can be downloaded at: https://www.mdpi.com/article/10.3390/jcm15207887/s1. Literature Search Strategy.

## Abbreviations

The following abbreviations are used in this manuscript:

BAT	Burnout Assessment Tool
BREATHE	Burnout Reduction: Enhanced Awareness, Tools, Handouts, and Education
CBT	Cognitive Behavioral Therapy
EEG	Electroencephalography
IL	Interleukin
MBI(-GS)	Maslach Burnout Inventory (-General Survey)
MBSR	Mindfulness-Based Stress Reduction
OLBI	Oldenburg Burnout Inventory
PFI	Professional Fulfillment Index
PSS	Perceived Stress Scale
RCT	Randomized Controlled Trial
RISE	Resilience, Integration, Self-awareness, Engagement
VR	Virtual Reality
