# Supplementary material for: Intensive Interventions for Burnout, Stress and Occupational Well-Being: A Critical Review and a Delivery-Format Framework
Source: J Clin Med. 2026 Oct 13;15(20):7887. doi: 10.3390/jcm15207887 (PMC13706483; doi:10.3390/jcm15207887)
Supplement: Supplementary file 1 [file jcm-15-07887-s001.zip › jcm-4522408-supplementary.pdf]

Supplementary Information for

Intensive Interventions for Burnout, Stress and Occupational Well-Being: A Critical Review and a Delivery-Format Framework

Anne Marie Novak and Shahar Lev-Ari

Literature Search Strategy

A comprehensive literature search was conducted across three databases (PubMed, APA PsycInfo, and Google Scholar) to identify peer-reviewed articles published between January 1, 2000, and March 31, 2026. The search utilized a combination of Boolean operators linking two primary concept blocks: target outcomes (burnout, occupational well-being) and intervention delivery formats (immersive, intensive, retreat).

Database-Specific Search Strings

Database	Search String
**PubMed**	(burnout[Title/Abstract] OR stress[Title/Abstract] OR "well-being"[Title/Abstract] OR wellbeing[Title/Abstract] OR exhaustion[Title/Abstract] OR "emotional exhaustion"[Title/Abstract] OR cynicism[Title/Abstract] OR disengagement[Title/Abstract] OR resilience[Title/Abstract] OR "psychological well-being"[Title/Abstract] OR "mental well-being"[Title/Abstract]) AND (intensive*[Title/Abstract] OR immersive[Title/Abstract] OR immersion[Title/Abstract] OR retreat*[Title/Abstract] OR residential[Title/Abstract] OR "non-residential"[Title/Abstract] OR nonresidential[Title/Abstract] OR workshop*[Title/Abstract] OR bootcamp*[Title/Abstract] OR "boot camp"[Title/Abstract] OR "short-term"[Title/Abstract] OR "brief intensive"[Title/Abstract] OR "multi-day"[Title/Abstract] OR "multi day"[Title/Abstract] OR "high-intensity"[Title/Abstract] OR "high intensity"[Title/Abstract] OR condensed[Title/Abstract] OR synchronous[Title/Abstract] OR "live online"[Title/Abstract])
**APA PsycInfo**	(TI(burnout OR stress OR "well-being" OR wellbeing OR exhaustion OR "emotional exhaustion" OR cynicism OR disengagement OR resilience OR "psychological well-being" OR "mental well-being") OR AB(burnout OR stress OR "well-being" OR wellbeing OR exhaustion OR "emotional exhaustion" OR cynicism OR disengagement OR resilience OR "psychological well-being" OR "mental well-being")) AND (TI(intensive* OR immersive OR immersion OR retreat* OR residential OR "non-residential" OR nonresidential OR workshop* OR bootcamp* OR "boot camp" OR "short-term" OR "brief intensive" OR "multi-day" OR "multi day" OR "high-intensity" OR "high intensity" OR condensed OR synchronous OR "live online") OR AB(intensive* OR immersive OR

Database	Search String
	immersion OR retreat* OR residential OR "non-residential" OR nonresidential OR workshop* OR bootcamp* OR "boot camp" OR "short-term" OR "brief intensive" OR "multi-day" OR "multi day" OR "high-intensity" OR "high intensity" OR condensed OR synchronous OR "live online"))
**Google Scholar**	"burnout" AND (intensive OR immersive OR retreat OR residential OR workshop)<br>"burnout" AND ("multi-day" OR "multi day" OR "short-term" OR "high-intensity" OR condensed)<br>("stress" OR "well-being" OR wellbeing) AND (intensive OR immersive OR retreat OR residential)<br>(resilience OR "psychological well-being") AND (intensive OR immersive OR retreat OR workshop)<br>("occupational burnout" OR "work-related stress") AND (retreat OR residential OR intensive OR immersive)<br>(burnout OR stress OR "well-being") AND (synchronous OR "live online") AND (intensive OR immersive)<br>(burnout OR stress OR resilience) AND (bootcamp OR "boot camp")

Study Selection and Screening Process

Following the execution of the searches, all identified citations were exported to reference management software (SciWheel) where duplicates were removed. The screening process proceeded in two stages. First, titles and abstracts were reviewed against the predefined inclusion and exclusion criteria. Articles appearing to meet the criteria, or those where eligibility was unclear from the abstract alone, were retrieved for full-text review.

Reviewer Roles and Disagreement Resolution

The primary investigator (A.M.N.) conducted the initial full-text review to confirm final eligibility based on the temporal-compression definitions established in the framework. A 20% random sample of the full texts was independently co-reviewed by the second author (S.L.-A.) to ensure consistent application of the inclusion criteria. Any disagreements regarding study inclusion or the classification of an intervention's delivery format were resolved through synchronous discussion between the two authors until consensus was reached by mapping the intervention parameters directly onto the four-dimension immersion framework.

Quality Assessment and Adjacent Evidence Distinction

Because this manuscript was conducted as a critical narrative review, a formal quantitative risk of bias assessment was not performed; instead, methodological limitations were narratively appraised within the text. Furthermore, to maintain a boundary between core qualifying studies and conceptual literature, virtual synchronous programs are strictly classified as adjacent evidence. No virtual studies met the temporal-

compression criteria required for the primary data tables, and this literature is incorporated exclusively within the discussion to approximate dismantling evidence.

ClinicalTrials.gov Search Strategy

To identify registered, ongoing, or unpublished trials of synchronous virtual intensives that meet the framework's inclusion criteria, a supplementary search of ClinicalTrials.gov was conducted. To capture the broadest possible range of interventions without being constrained by specific condition terminology, three separate expanded queries were executed across all study statuses.

1. **Query 1 (Digital & Condensed Format):**
    a. Condition/Disease: Burnout OR Occupational Stress OR Compassion Fatigue
    b. Other Terms: (virtual OR online OR digital OR remote OR videoconference OR web-based OR internet) AND (intensive OR retreat OR workshop OR bootcamp OR immersive OR seminar OR weekend OR "single session" OR accelerated)

2. **Query 2 (Professional Well-Being):**
    a. Condition/Disease: Well-being OR Resilience OR Occupational Health
    b. Other Terms: (virtual OR online) AND (retreat OR intensive OR bootcamp OR workshop)

3. **Query 3 (Profession-Specific Target):**
    a. Condition/Disease: [Left Blank]
    b. Other Terms: (physician OR nurse OR teacher OR educator OR "healthcare worker" OR employee) AND (virtual OR online OR remote) AND (retreat OR bootcamp OR intensive)
